# Identification and dynamic quantification of regulatory elements using total RNA

**DOI:** 10.1101/gr.253492.119

**Published:** 2019-11

**Authors:** Sascha H. Duttke, Max W. Chang, Sven Heinz, Christopher Benner

**Affiliations:** Department of Medicine, University of California, San Diego, La Jolla, California 92093, USA

## Abstract

The spatial and temporal regulation of transcription initiation is pivotal for controlling gene expression. Here, we introduce capped-small RNA-seq (csRNA-seq), which uses total RNA as starting material to detect transcription start sites (TSSs) of both stable and unstable RNAs at single-nucleotide resolution. csRNA-seq is highly sensitive to acute changes in transcription and identifies an order of magnitude more regulated transcripts than does RNA-seq. Interrogating tissues from species across the eukaryotic kingdoms identified unstable transcripts resembling enhancer RNAs, pri-miRNAs, antisense transcripts, and promoter upstream transcripts in multicellular animals, plants, and fungi spanning 1.6 billion years of evolution. Integration of epigenomic data from these organisms revealed that histone H3 trimethylation (H3K4me3) was largely confined to TSSs of stable transcripts, whereas H3K27ac marked nucleosomes downstream from all active TSSs, suggesting an ancient role for posttranslational histone modifications in transcription. Our findings show that total RNA is sufficient to identify transcribed regulatory elements and capture the dynamics of initiated stable and unstable transcripts at single-nucleotide resolution in eukaryotes.

Transcription decodes the regulatory signals inscribed in the genome to initiate gene expression in response to cellular or external cues. At its heart lies the transcription start site (TSS), where RNA polymerase II starts gene transcription. Transcriptional regulators bind to specific DNA sequences near the TSS to remodel chromatin and recruit the molecular complexes necessary to start transcription. Annotation of genes and regulatory elements and the analysis of the underlying molecular mechanisms regulating transcription therefore depend on the identification of TSSs and the measurement of their activity genome-wide.

The advent of nascent RNA sequencing methodologies has revealed a plethora of unstable transcripts. Such transcripts arise from divergent transcription of promoter regions (Core et al. 2008; Preker et al. 2008; Seila et al. 2008; Neil et al. 2009), antisense transcription (Berretta and Morillon 2009), and, particularly in mammals, transcription initiation from enhancers (De Santa et al. 2010; Kim et al. 2010). Although the biological function of these transient RNAs is debated, enhancer RNAs (eRNAs) reveal active enhancers (Wang et al. 2011), and eRNA expression levels correlate with nearby gene expression (Hah et al. 2013; Cheng et al. 2015; Azofeifa et al. 2018; Mikhaylichenko et al. 2018). Enhancers are critical modulators of gene activity and integrate spatiotemporal cues to coordinate cell-type–specific gene expression. Compared to promoters, enhancers are enriched for cell lineage-determining transcription factor binding sites. Mapping active enhancers is therefore key to deciphering regulatory networks and cell-type–specific gene expression. To avoid confusion in this study, we will refer to enhancers as “distal regulatory elements” as they were defined by transcription and active chromatin marks, rather than physiological functionality. Other unstable RNAs include diverse precursor RNAs, such as pri-miRNAs that avoid detection owing to being rapidly processed into their mature forms such as miRNAs (Lee et al. 2002). Furthermore, the process of transcription rather than the RNA itself has been shown to impact genome conformation (Heinz et al. 2018), DNA topology (Teves and Henikoff 2014), and chromatin states (Santos-Rosa et al. 2002). It is therefore critical to assay active promoter and distal regulatory elements in a quantitative and sensitive manner when investigating biological phenomena, gene regulation, or regulatory networks.

RNA stability presents a continuum spanning RNA half-lives of less than a minute to several hours, which impacts their detection (Wada and Becskei 2017). Unstable RNAs and their initiation sites are difficult to identify with methods that capture steady-state RNA levels such as conventional RNA-seq or 5′ 7-methylguanosine cap (5′ cap)–enriched RNA sequencing methods such as 5′RNA-seq or CAGE (Shiraki et al. 2003). In contrast, methods that capture nascent RNA detect transcripts and their TSSs independent of their stability. These methods include using nuclear or chromatin run-on reactions with modified nucleotides to isolate nascent transcripts (GRO-seq [Core et al. 2008], PRO-seq [Kwak et al. 2013], ChRO-seq [Chu et al. 2018]), sequencing RNA polymerase-associated RNAs (NET-seq; Churchman and Weissman 2011), in vivo labeling RNA and enrichment of newly synthesized RNA (Salic and Mitchison 2008; Duffy et al. 2015; Schwalb et al. 2016), or depletion of cellular components to deter the degradation of unstable RNAs (Preker et al. 2008; Davidson et al. 2019). These methods faithfully map transcribed regulatory elements and reveal the transcriptome at an unprecedented scale. However, the requirement of nuclei isolation, pulse labeling during cell culture, or genetic manipulations prevents their application to tissues, frozen samples, or nonmodel organisms. Furthermore, as the sequencing reads from these assays largely align to gene body regions, they are useful for defining regulatory elements, transcription units, or rates but lack the positional resolution to precisely locate TSSs (Danko et al. 2015; Azofeifa and Dowell 2017). Assays that combine 5′ cap enrichment (Maruyama and Sugano 1994) with run-on sequencing such as 5′GRO-seq/GRO-cap (Kruesi et al. 2013; Lam et al. 2013) concentrate reads at the TSS of regulatory elements and enable (semi-)quantitative assessment of transcription initiation rates at single-base resolution. This enables the identification of TSS of both stable (protein-coding and noncoding RNAs) and unstable transcripts (eRNAs, divergent transcripts) at unpreceded scale and reveals active regulatory elements genome-wide (Core et al. 2014). However, although 5′GRO-seq is feasible in primary tissues (Hetzel et al. 2016), it is laborious and difficult to scale up. It is further unclear how far the actual representation of cell types is maintained during isolation procedures as, for example, the sensitivity of distinct mammalian cells types to the detergents or osmotic imbalance varies more than 10-fold.

It was previously shown that sequencing newly initiated RNA polymerase II transcripts can accurately define stable and unstable TSSs (Preker et al. 2008; Seila et al. 2008; Affymetrix/Cold Spring Harbor Laboratory ENCODE Transcriptome Project 2009; Lister et al. 2009; Nechaev et al. 2010; Gu et al. 2012; Scruggs et al. 2015). These transcripts can be enriched by selecting small RNAs with 5′ cap and 3′ OH that are shorter than those native to the steady-state RNA polymerase II transcriptome. Inspired by established small RNA-seq methods including Start-seq (Nechaev et al. 2010) and CapSeq (Gu et al. 2012), we have developed a protocol we termed capped-small RNA-seq (csRNA-seq) that captures these short TSS-associated transcripts from total RNA (Fig. 1A,B; Supplemental Fig. S1A,B). Using total RNA as input to reliably determine the TSS of promoters and distal regulatory elements at single-nucleotide resolution enables accurate annotation of genes and regulatory elements and the study of dynamic gene regulation and regulatory networks in any fresh or frozen eukaryotic sample or tissue from which total RNA can be extracted.

**Figure 1.**
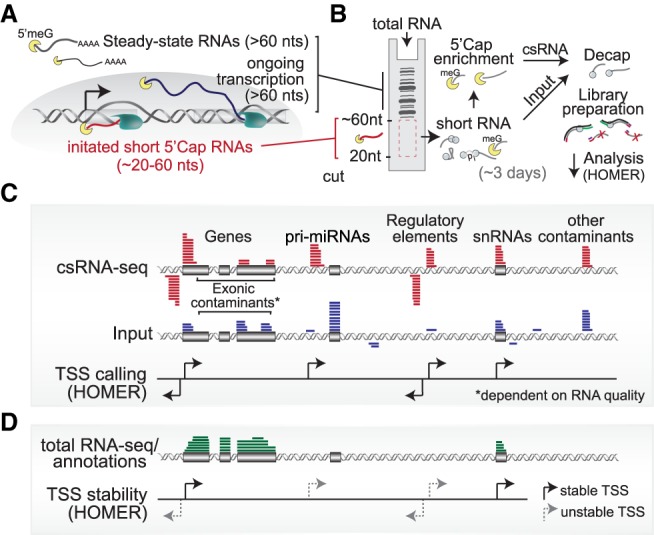
Overview of capped small RNA-seq. (*A*) Schematic of short initiated RNAs that are captured by csRNA-seq and (*B*) a graphical depiction of the method starting from total RNA. (*C*) Transcription start site (TSS) clusters are determined through the enrichment of small capped RNAs over the total small RNA input using HOMER. The schematic shows the typical distribution of csRNA-seq and input at various genomic features. (*D*) Integration of genome annotations or total RNA-seq enables the assignment of TSSs to stable and unstable transcripts.

## Results

### csRNA-seq accurately captures initiated stable and unstable RNAs from total RNA

Sequencing capped, small RNAs from total RNA as starting material enables the study of a wide variety of samples. However, degradation products of highly abundant RNAs and short noncapped RNAs can give rise to false-positive TSS signals, especially when RNA is extracted from banked tissues or samples collected in the field. To relax the requirement of quality RNA and computationally identify and exclude false-positive TSS calls, total small RNA input libraries that include uncapped RNAs are also profiled. csRNA-seq determines TSS clusters by the relative enrichment of capped small RNAs over the total input (Fig. 1C; Supplemental Fig. S1C–E). By using this approach, we were able to define TSSs from csRNA-seq libraries generated from highly fragmented RNA with RINs as low as two. In addition to controlling for degradation-induced artifacts, input libraries represent a resource for discovery as they capture all small uncapped RNAs, including microRNAs, Piwi-interacting RNAs (piRNAs), small interfering RNAs (siRNAs), and other small, processed RNAs present (Supplemental Fig. S1B,F). Ribosomal RNA–depleted RNA-seq or genome annotations can be integrated to further limit false-positive TSS clusters found in highly expressed exons (Supplemental Fig. S1E) and to assign stable and unstable transcript status to TSS clusters (Fig. 1D). This assignment of RNA stability facilitates distinguishing gene promoters from distal regulatory elements such as enhancers. Of note, csRNA-seq and matched RNA-seq data can also be used to generate accurate de novo genome annotations. To facilitate simple and accurate TSS cluster discovery and annotation from csRNA-seq and control data, we developed a software analysis framework that has been integrated into the HOMER software suite (Heinz et al. 2010).

**Figure 2.**
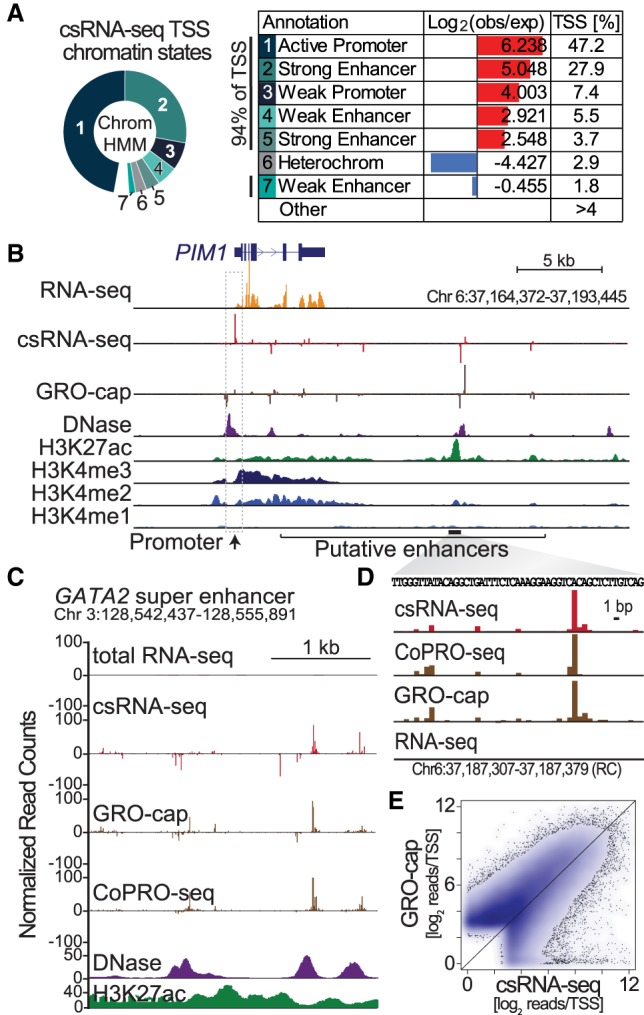
csRNA-seq accurately captures stable and unstable sites of transcription initiation sites from total RNA. (*A*) Chromatin states of csRNA-seq TSS clusters in human K562 cells as determined by ChromHMM. (*B*) Comparison of csRNA-seq TSS with other genome-wide assays at the *PIM1* locus. (*C*) Example of an unstable TSS cluster from a gene-distal regulatory element at single-nucleotide resolution. (*D*) Comparison of read depths at TSS clusters determined by csRNA-seq and GRO-cap (Core et al. 2014) in K562 cells.

To evaluate the sensitivity and reproducibility of csRNA-seq, we generated duplicate csRNA-seq and small RNA input libraries using 10 µg of total RNA from separate cultures of human K562 myelogenous leukemia cells (Supplemental Fig. S2A,B). csRNA-seq data are highly consistent and quantitatively reproducible across independent replicate experiments (*r* = 0.91) (Supplemental Fig. S2A). Sequencing csRNA-seq libraries to a depth of approximately 15 million reads efficiently covered regulatory features in the human genome (Supplemental Fig. S2C) and identified 54,000 candidate TSS clusters. Comparing these csRNA-defined TSS clusters with existing annotations and other data generated in K562 cells revealed a global overlap with known features of transcription initiation (Supplemental Fig. S3A). Ninety-four percent of the TSSs mapped to promoter or enhancer regions as defined by ChromHMM (Fig. 2A; Supplemental Fig. S2D; Ernst and Kellis 2012), and >92% of TSS clusters overlapped DNase-hypersensitive regions (Thurman et al. 2012). csRNA-seq accurately identified the TSSs of known genes and transient RNAs (Fig. 2B), including pre-miRNAs (Supplemental Fig. S1F) as well as distal regulatory elements such as putative eRNAs in super-enhancers (Fig. 2C) at single-nucleotide resolution (Fig. 2D). The ability of csRNA-seq to identify TSS was dependent on the expression level of transcripts from each locus. With expression levels greater than 4 FPKM, TSSs for >90% of genes were identified, in some cases providing novel TSS annotation (Supplemental Fig. S2E,F).

Initiated transcript profiles generated by csRNA-seq bear a resemblance to nascent initiation profiles generated by GRO-cap in K562 cells (Core et al. 2014). TSS cluster locations and preferred nucleotide frequencies relative to the TSSs were highly concordant between the methods (Fig. 2B; Supplemental Figs. S2D, S3B). Transcript initiation levels among csRNA-seq and GRO-cap were overall correlated (*r* = 0.61) (Fig. 2E) with 78% of the identified TSS clusters shared among csRNA-seq and GRO-cap. The primary TSSs from both methods started from YR dinucleotides (Supplemental Fig. S3C) with a strong preference for A at the +1 site and the canonical Initiator motif (Smale and Baltimore 1989; Vo Ngoc et al. 2017). Method-specific TSSs were preferentially found at distal regulatory elements (Supplemental Fig. S2D, S3D) and had lower levels of nascent transcription, open chromatin, H3K27ac, and RNA polymerase II recruitment relative to TSSs identified by both methods (Supplemental Fig. S3E). Clusters specific to csRNA-seq were more frequently found at small nuclear RNA genes (i.e., snRNA) and correspondingly enriched for a PSE motif (Wirth et al. 1987). GRO-cap–specific clusters were enriched for the motif of the bZIP transcription factor DNA damage inducible transcript 3 (DDIT3, also known as CHOP) (Supplemental Fig. S3F). Most of these observations suggest that the differences in TSSs called by either method might be more reflective of laboratory-specific differences (subclone or cell culture conditions) than the technical differences between the methods. Together these data show that csRNA-seq captures the TSSs of active promoters and distal regulatory elements with high fidelity and accuracy, bearing a high degree of similarity to profiles derived from nascent transcription initiation techniques.

### csRNA-seq captures TSSs of rapidly degraded transcripts

**Figure 3.**
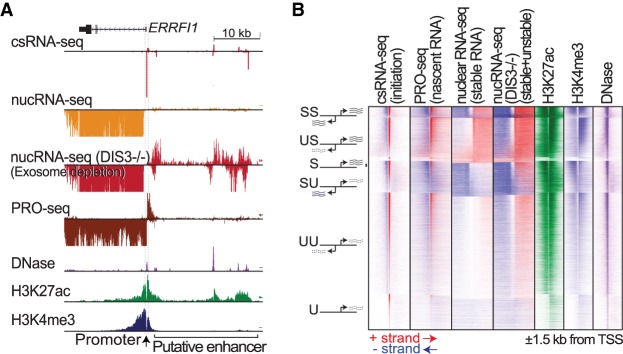
csRNA-seq captures the initiation of transient transcripts rapidly degraded by the exosome. (*A*) Comparison of csRNA-seq with nuclear RNA-seq from wild-type and exosome-depleted HCT116 cells at the *ERRFI1* locus (Chr 1: 8,008,489–8,051,491). (*B*) Global comparison of csRNA-seq with data from the nascent RNA-seq method PRO-seq, as well as wild-type and exosome-depleted nuclear RNA-seq and chromatin profiling (DNase, H3K27ac, H3K4me3) in HCT116 cells.

The transcriptome encodes an abundance of short-lived transcripts that are rapidly degraded by the DIS3 exoribonuclease component of the exosome (Szczepińska et al. 2015; Davidson et al. 2019). Sensitive identification of these transcripts and their TSSs usually requires live cells to isolate nuclear RNA or nuclear run-on products (Core et al. 2008, 2014; Nechaev et al. 2010; Lam et al. 2013). To test whether these transcripts can be readily detected from only total RNA, we performed csRNA-seq in HCT116 cells, for which RNA-seq data for both DIS3 exoribonuclease degradation (Davidson et al. 2019) and nascent transcription (PRO-seq) (Rao et al. 2017) are available for comparison. TSSs were called and stability of the associated transcripts was inferred by integrating csRNA-seq and total RNA-seq data. This analysis indicated that only 40% of the 69,000 total TSSs identified initiated stable transcripts in HCT116 cells. As exemplified by the *ERRFI1* locus (Fig. 3A) and summarized genome-wide for all TSSs (Fig. 3B), both transient and stable transcript TSSs are accurately captured by csRNA-seq. Stable transcripts displayed evidence for RNA-seq reads downstream from the TSSs in control and exosome-depleted samples, whereas unstable transcripts only became detectable by RNA-seq under exosome-depleted conditions. The initiation sites of stable and unstable RNAs exhibit considerable overlap with respect to chromatin architecture and epigenetic modifications (Supplemental Fig. S4; Core et al. 2014), with histone 3 lysine 4 trimethylation (H3K4me3) being a major indicator of transcript stability (Santos-Rosa et al. 2002; Heintzman et al. 2007; Duttke et al. 2015). In line with these previous findings, histone modifications associated with activation (H3K27ac/H3K4me3) accumulated directly downstream from the TSSs in a manner dependent on the direction of transcription and stability of the transcribed RNA (Fig. 3B; Supplemental Fig. S4). These results substantiate that analysis of total RNA by csRNA-seq combined with RNA-seq can be used to profile stable and unstable transcripts and identify their cognate TSSs.

### csRNA-seq identifies cell-type–specific gene regulatory elements and their underlying transcription factor networks

**Figure 4.**
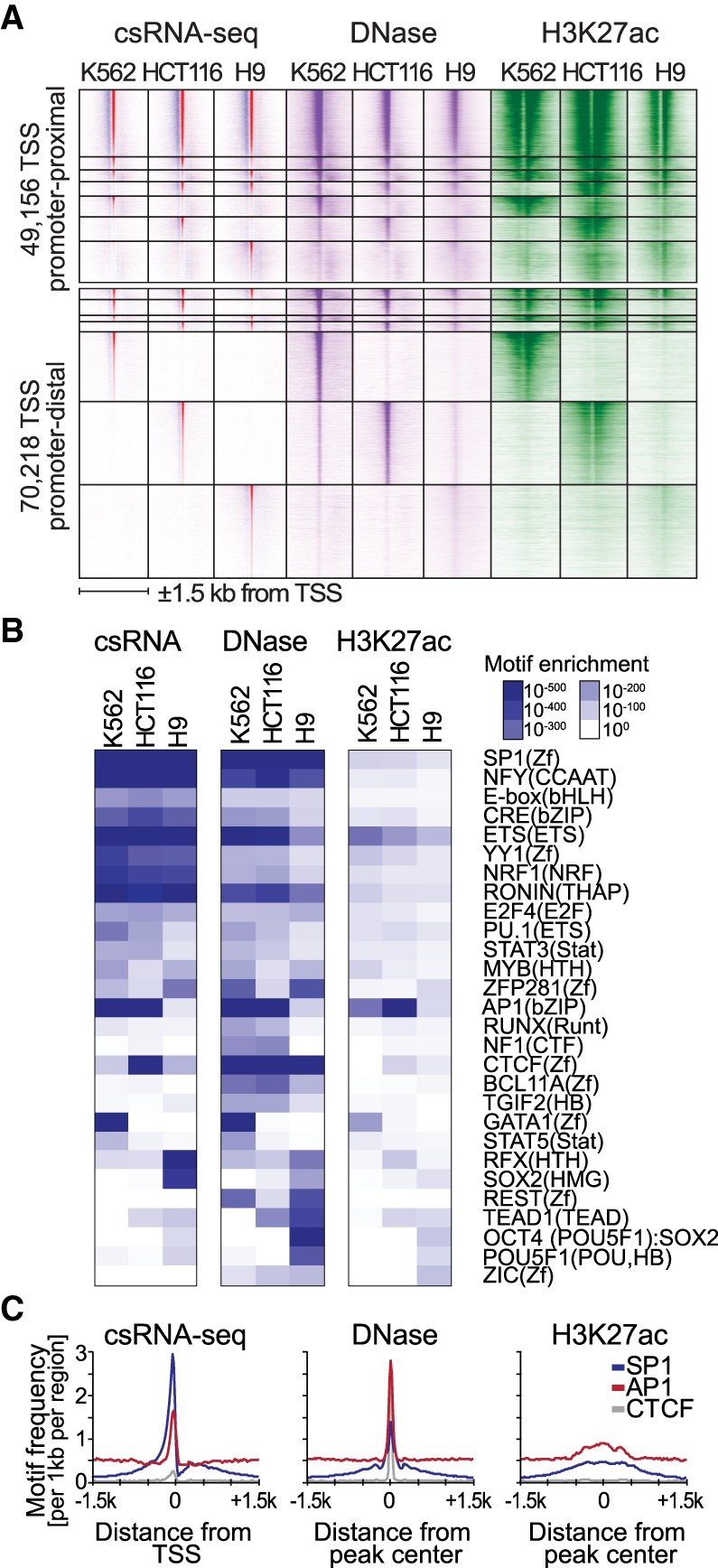
csRNA-seq identifies active promoters and distal regulatory elements and their underlying transcription factor networks in a cell-type–specific manner. (*A*) Grouping of common and cell-type–specific csRNA-seq TSSs with DNase-seq and H3K27ac ChIP-seq across three different human cell lines (±1.5 kb to the TSS). (*B*) Known DNA motifs enriched in the distal regulatory elements of human K562, HCT116, and H9 embryonic stem cells identified using HOMER. Motif enrichment was calculated for sites located within (−150,+50) relative to TSSs for csRNA-seq or from (−100,+100) or (−500,+500) relative peak centers for DNase-seq and H3K27ac ChIP-seq, respectively. (*C*) TSSs identified by csRNA-seq provide a single-nucleotide anchor that facilitates accurate spatial analysis of DNA motifs compared with peaks as defined by DNase-seq or H3K27ac ChIP-seq.

Cell identity is informed by distal regulatory elements that in concert with promoters drive cell-type–specific gene expression (Maston et al. 2006; The ENCODE Project Consortium 2012). To better understand gene regulation in health and disease, it is critical to accurately define active promoters and distal regulatory elements to decode the underlying transcription factors motifs and other features that ultimately drive gene expression. ChIP-seq for histone modifications associated with gene activation (e.g., H3K27ac) or open chromatin profiling (DNase-seq or ATAC-seq) (Buenrostro et al. 2013) are the most commonly used methods to globally profile regulatory regions. Alternatively, active regulatory elements can be directly determined by transcription (De Santa et al. 2010; Kim et al. 2010; Wang et al. 2011). To assess the utility of csRNA-seq to decode the “transcriptional regulome,” we defined promoter-proximal and distal TSSs across three distinct human cell lines: K562 myelogenous leukemia cells, HCT116 colon cancer cells, and H9 embryonic stem cells. A comparison of approximately 130,000 total nonredundant TSS clusters revealed common and unique usage patterns across different cell types. Consistent with previous findings (Heinz et al. 2010; The ENCODE Project Consortium 2012), the greatest cell-type–specific variation in activity occurred at distal regulatory elements. At these sites, TSS usage measured by csRNA-seq closely matched patterns of common and cell-type–specific H3K27ac enrichment and DNase hypersensitivity (Fig. 4A).

We next performed DNA motif analysis using HOMER (Heinz et al. 2010) to probe transcription factor motifs enrichment near TSS [−150,+50] for each cell type. Motifs recognized by ubiquitous transcription factors typically present at gene promoters (i.e., SP1, NFY) were strongly enriched at TSSs common to all three cell types. In contrast, motifs corresponding to lineage-specific transcription factors were selectively enriched near TSSs specifically transcribed in the appropriate cell types: GATA motifs were found in myelogenous leukemia K562 cells (Shimizu et al. 2008); SOX2, RFX, and OCT4 (POU5F1):SOX2 composite motifs were confined to H9 embryonic stem cells (Poletti et al. 2015); AP1 binding sites were common to epithelial colon cancer cell line HCT116 and K562 cells; and the CTCF motif was enriched in HCT116 cells (Fig. 4B). These results accentuate that csRNA-seq can accurately define active regulatory elements and characterize the associated DNA sequence motifs across different cell types.

To probe the fidelity of csRNA-seq in decoding the regulome, we next performed DNA motif enrichment analysis in DNase-seq and H3H27ac ChIP-seq peak regions, which yielded a similar set of motifs and successfully identified the appropriate lineage-specific motifs for each cell type (Fig. 4B). Motif enrichment was generally weaker for H3K27ac owing to the lower spatial resolution of the assay (∼1 kb vs. ∼200 bp for csRNA/DNase) (Fig. 4C); however, the overall motif enrichment pattern closely followed the results from csRNA-seq TSSs. The similarity is underscored by the fact that H3K27ac and csRNA-seq signals are correlated and identify similar regions of the genome (Supplemental Fig. S5A), consistent with histone acetylation being closely associated with transcription (Stasevich et al. 2014). DNase-seq peaks exhibited several motifs distinct from those enriched in csRNA-seq and H3K27ac, including the repressor REST, the architectural factor CTCF, and several C_2_H_2_-type zinc finger transcription factors. It is important to note that not every open chromatin region is actively transcribed (Supplemental Fig. S5B; Natarajan et al. 2012), suggesting csRNA-seq could be used in combination with DNase-seq to effectively annotate inactive but accessible regulatory elements to identify transcription factors associated with repression or other molecular functions. Direct definition of transcriptional activity at base resolution further enables analyzing DNA motifs in a distance-specific and directional manner relative to the TSSs, revealing positional motif preferences relative to transcription initiation (Fig. 4C). In summary, direct identification of active regulatory elements from csRNA-seq TSSs reveals cell-type–specific gene expression and *cis*-regulatory elements with high accuracy and facilitates downstream analysis such as investigating the architecture underlying transcription initiation or identification of enriched transcription factors motifs.

### csRNA-seq sensitively quantifies changes in transcription initiation

**Figure 5.**
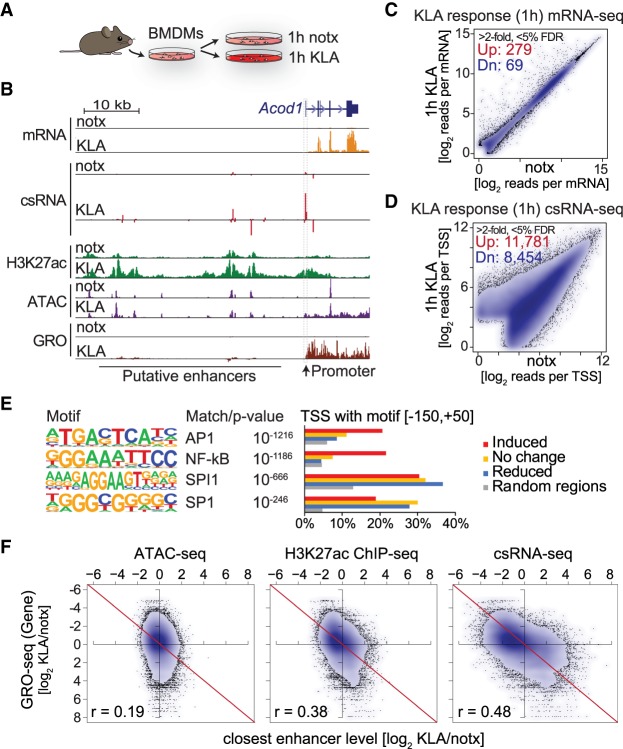
csRNA-seq captured changes in the transcriptome with high fidelity. (*A*) Bone marrow–derived macrophages were isolated from C57Bl6 mice and stimulated with KLA (TLR4 agonist) for 1 h. (*B*) Comparison of transcriptional and epigenetic profiling methods at the mouse *Acod1* locus in untreated (Ctrl) and activated (KLA) conditions after stimulation for 1 h with KLA. (*C*) Differentially expressed features in response to 1-h KLA as captured by RNA-seq and (*D*) csRNA-seq (348 vs. 20235 features at greater than twofold difference and <5% FDR). (*E*) DNA motifs enriched in KLA-induced regulatory regions compared with random, reduced, or unaltered regions (−150,+50 relative to TSSs). (*F*) Comparison of ATAC-seq, H3K27ac, and csRNA in capturing alterations is distal regulatory elements relative to changes in nearby gene transcription upon 1-h KLA stimulation. Scatter plots show the log_2_ ratio of changes in activity upon KLA stimulation in distal regulatory elements relative to the change in gene expression of the nearest expressed gene as captured by GRO-seq.

To assess the ability of csRNA-seq to quantitatively evaluate changes in gene expression, we profiled murine bone marrow–derived macrophages (BMDMs) activated by the TLR4 agonist Kdo2-lipid A (KLA) (Fig. 5A; Raetz et al. 2006). csRNA-seq faithfully captured changes in transcription initiation at activated response genes and their distal regulatory elements after 1 h of stimulation with KLA (Fig. 5B). Compared with RNA-seq from the same samples that identified 279 induced and 69 down-regulated genes (Link et al. 2018), csRNA-seq captured 11,781 up- and 8454 down-regulated TSS clusters (greater than twofold, FDR <5%) (Fig. 5C,D; Supplemental Fig. S5C). A vast majority of regulated csRNA-seq TSSs were associated with unstable transcripts (88%) located at promoter-distal regulatory elements (71%). Although the function of many of these transcripts is speculative (Wu and Sharp 2013; Marchese et al. 2017), eRNA transcription is highly predictive of transcription factor activity and transcriptional networks (Wang et al. 2011; Hah et al. 2013; Cheng et al. 2015; Azofeifa et al. 2018). De novo motif analysis of induced TSSs with HOMER recovered strong enrichment for motifs bound by transcription factors AP-1 and NF-κB (Fig. 5E), which mediate the primary KLA response (Fujioka et al. 2004).

To assess if quantitative changes in transcription initiation at putative enhancer regions are predictive of regulation of nearby genes, we compared KLA-induced changes in csRNA-seq, H3K27ac, and ATAC-seq at distal regulatory elements with those of the nearest expressed gene as quantified by GRO-seq (Link et al. 2018). This analysis shows that csRNA-seq has the highest predictive power for linking activation of distal regulatory elements to proximal genes (Pearson's *r* = 0.48), followed by H3K27ac ChIP-seq (Pearson's *r* = 0.38) and ATAC-seq signal (Pearson's *r* = 0.19). Additionally, csRNA-seq displayed a fourfold higher dynamic range than ATAC-seq or H3K27ac ChIP-seq (Fig. 5F). These differences are exemplified by the putative enhancers upstream of the *Acod1* locus (Fig. 5B). Consistent with previous findings (Kaikkonen et al. 2013), many of these putative enhancers already exhibit open chromatin, low levels of transcription, and H3K27ac in untreated cells. Upon stimulation, strong induction of transcription initiation at these sites is only sometimes associated with further increases in chromatin accessibility. Changes in csRNA-seq and H3K27ac are more closely correlated, with changes in H3K27ac most prominent just downstream from regulated TSSs (Supplemental Fig. S5E). Together, these findings establish csRNA-seq as a highly sensitive method to quantify changes in transcription initiation at both promoters and distal regulatory elements from total RNA to study gene regulatory networks.

### csRNA-seq captures stable and unstable initiated transcription across eukaryotic specimens and tissues

**Figure 6.**
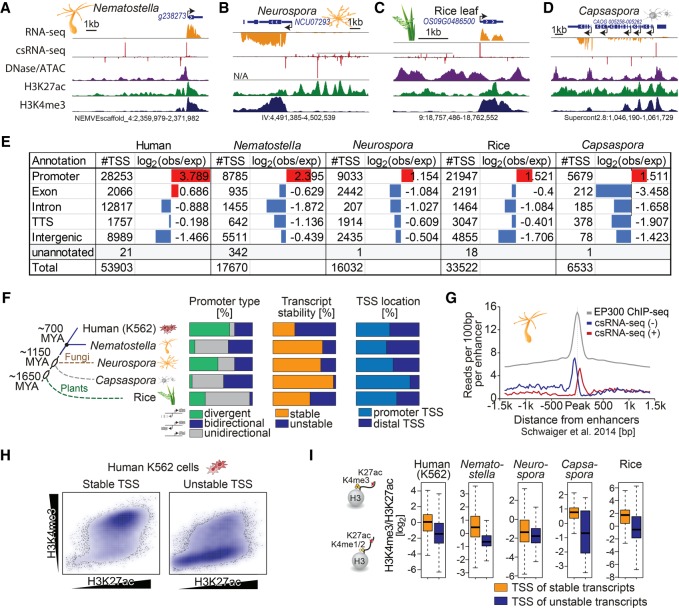
csRNA-seq accurately profiles TSSs across eukaryotes. Capturing stable and unstable transcripts across eukaryotes by csRNA-seq reveals a gradual increase in unstable transcripts as more complex body plans evolved and conserved roles for the histone modifications H3K4me3 and H3K27ac. Example loci from diverse eukaryotes from across the kingdoms (*A*) *Nematostella* (metazoa), (*B*) *Neurospora* (fugi), (*C*) rice (plants), and (*D*) *Capsaspora* (protist). (*E*) Comparison of where TSSs defined by csRNA-seq are relative to genome annotations. (*F*) Species dendrogram with approximate divergence time and diagram of the percentage of stable versus unstable and unidirectional versus bidirectional transcripts. (*G*) csRNA-seq reads centered on the *Nematostella* enhancer regions defined by Schwaiger et al. (2014). (*H*) Scatterplot of H3K4me3 versus H3K27ac levels for stable and unstable transcripts from human K562 cells. (*I*) Boxplot with the log_2_ ratio of H3K27ac/H3K4me3 for the TSSs of stable and unstable transcripts.

By capturing stable and unstable transcripts from total RNA, csRNA-seq overcomes limitations of methods that require nuclei isolation or other manipulations that are difficult in nonmodel organisms or tissues. This advance enables the characterization of the initiating transcriptome in any fresh or frozen eukaryotic sample or tissue for which total RNA can be extracted. To illustrate this, we profiled the starlet anemone *Nematostella vectensis* (metazoa), the fungus *Neurospora crassa*, rice plant leaves (*Oryza sativa*), and the protist *Capsaspora owczarzaki* (Sebé-Pedrós et al. 2016). These species were selected to broadly cover the evolutionary tree of life as well as to show the feasibility of csRNA-seq in samples were morphological constraints and/or secondary metabolites hinder fixation or nuclei isolation. Captured TSSs predominantly mapped to DNase-sensitive nucleosome-free regions bordered by H3K27-acetylated nucleosomes in each species (Fig. 6A–D; Supplemental Fig. S6A). Across the species, TSSs were enriched at annotated promoters and underrepresented within gene bodies (Fig. 6E; Supplemental Fig. S6B). The nucleotide frequency preferences near TSS showed a strong preference for the Initiator motif and prominent TATA signature in plants and metazoa (Supplemental Fig. S6C). These data show how csRNA-seq captures initiating transcripts across diverse eukaryotic samples and tissues and thereby open up new avenues and organisms to study.

### Ancient roles for H3K27ac and H3K4me3 in eukaryotes

The regulatory innovations leading to the evolution of more derived body plans are largely speculative. Multicellular life evolved several times independently (e.g., Grosberg and Strathmann 2007), and it is currently an open question to what extent the diverse unicellular protists, fungi, plants, or early-branching animals share regulatory principles and architecture with bilaterians such as humans or *Drosophila*. Taking advantage of csRNA-seq and total RNA-seq profiling, we annotated TSS transcript stability and found significant variation in the prevalence of RNA stability and promoter types across species (Fig. 6F). Apart from the protist *Capsaspora*, a sizeable fraction of TSSs from each species initiated unstable transcripts, usually from gene-distal regulatory elements. For example, in the cnidarian *Nematostella*, unstable transcripts frequently originate from distal regions previously defined as enhancers (Schwaiger et al. 2014), suggesting these transcripts are eRNAs (Fig. 6A,G; Supplemental Fig. S6C). Similarly, unstable promoter-distal TSSs found in *Neurospora* and rice resemble bilaterian eRNAs, and many cluster in regions with highly transcribed genes, analogous to mammalian super-enhancers (Fig. 6B,C; Whyte et al. 2013). Similar to the situation in mammalian cells (Fig. 3B; Supplemental Fig. S4) and previous findings in *Drosophila* (Kharchenko et al. 2011; Duttke et al. 2015), H3K4me3-containing nucleosomes were uncommon at distal regulatory elements and largely confined to the start sites of stable RNAs throughout the analyzed eukaryotes (Fig. 6H,I). Given that these species span over 1.6 billion years of evolution (Parfrey et al. 2011), these data provide evidence that transcription initiation in distal regulatory elements likely evolved before the emergence of the Bilateria, and that the role of histone modifications H3K27ac and H3K4me3 with respect to transcription and transcript stability manifested early during eukaryotic evolution.

## Discussion

Here we introduce csRNA-seq to quantitatively capture initiated transcripts and define their TSSs directly from total RNA. The transcription initiation patterns at gene promoters and distal regulatory elements discovered by this approach are similar to the results of nascent RNA profiling methods (e.g., GRO-cap/5′GRO-seq) (Kruesi et al. 2013; Lam et al. 2013). TSSs defined by csRNA-seq are also highly correlated with Start-seq data, which uses nuclear RNA (Nechaev et al. 2010; Scruggs et al. 2015). Although isolating nuclear RNA removes degraded RNAs, noninitiated small RNAs (e.g., miRNA), and other abundant cytoplasmic RNA species, it had a minor impact on TSS identification and quantification (*r* = 0.77) (Supplemental Fig. S5D,F).

csRNA-seq identifies changes in activity at regulatory elements with higher dynamic range and better correlation with neighboring gene transcription changes than assays such as ATAC-seq or H3K27ac ChIP-seq. Furthermore, unlike these assays, csRNA-seq determines TSSs (akin to peaks) with single-nucleotide resolution. This precision boosts the sensitivity of motif finding approaches for identifying motifs for key lineage-determining and signal response transcription factors and enables accurate genome annotation. The fact that it uses total RNA as starting material makes csRNA-seq broadly applicable across eukaryotes. For example, csRNA-seq enables the characterization of transcripts in species in which physiological constraints such as cell walls and secondary metabolites or biosafety (e.g., crops, pathogenic fungi, or virus-infected tissues) hinder nuclei isolation for nascent RNA sequencing methods. Its focus on sequencing 5′ ends of initiated transcripts efficiently concentrates sequencing power to active promoters and enhancers, enabling the profiling of promoter and enhancer regulation in complex genomes such as humans with as few as 15 million single-end reads (Supplemental Fig. S2C).

At the same time, the 5′ bias of csRNA-seq reads make it unsuitable for tracking RNA polymerase II elongation or termination. Because transcripts captured by csRNA-seq are inherently short, the method does not allow the unique mapping of transposon-derived RNAs or other transcripts derived from highly repetitive regions. Likewise, csRNA-seq will be less effective for studies looking to quantify allele-specific expression. Another practical limitation of the assay is the requirement for a relatively large amount of starting material (∼10 µg of total RNA or approximately 5–10 million cells). Although we have generated libraries from <1 µg total RNA, sufficient starting material improves data quality and reproducibility.

csRNA-seq biochemically enriches for short RNAs with a 3′ hydroxyl group and a 5′-capped oligophosphodiester that protects them from dephosphorylation and exonuclease digest. However, the current selection of enzymes used in the csRNA-seq protocol do not distinguish alternative 5′ cap structures or other phosphodiester modifications such as adenylation. The requirement for such 5′ modifications may limit the use of csRNA-seq in some protists that lack canonical 5′ capping machinery (Shuman 2001). Depending on the RNA quality, input libraries are thus required to limit a possible bias from degraded fragments of abundant stable RNAs (Supplemental Fig. S6D). The ability to use total RNA as input allows researchers to source samples from around the world with minimal biosafety risk and at moderate costs. Exploiting this feature, we investigated stable and unstable RNAs across five eukaryotes that together span more than 1.6 billion years of evolution. This analysis revealed common themes among TSSs throughout evolution. We observed strong fluctuations in nucleotide frequencies near TSSs and a preference to initiate transcription from YR(+1) dinucleotides common to all five eukaryotic species (Supplemental Fig. S6C). Differences in TATA box usage between species and evidence for an expanded Initiator motif in *Capsaspora* suggest that core promoter sequence elements and their usage have diverged throughout evolution. The chromatin architecture at TSSs, with a nucleosome-depleted region centered on the proximal promoter flanked by nucleosomes with active histone modifications (e.g., H3K27ac), is similar between the eukaryotic species assayed (Supplemental Fig. S6A). The levels of H3K27ac found upstream of the TSSs are indicative of the levels of bidirectional transcription in each organism. Across evolutionarily distant eukaryotes, promoter-distal regions with unstable transcripts shared common features with mammalian enhancers, whereas unstable transcripts near promoters resembled promoter upstream transcripts (PROMPTs) (Preker et al. 2008). Clear evidence of (promoter-distal) enhancer transcription was observed in cnidarians, suggesting the mechanisms giving rise to eRNAs evolved before the split of the Bilateria, although similar loci with unstable TSSs identified in *Neurospora* and rice hint that eRNAs may have evolved much earlier. As more complex metazoan body plans emerged, the relative percentage and diversity of unstable transcripts increased. It is tempting to speculate that this increase in TSS diversity may be owing to a potentially higher demand for regulatory diversity as more and more cell types emerged.

Throughout the eukaryotic kingdoms, H3K27ac is associated with all active TSSs, whereas H3K4me3 is largely confined to the promoters of stable transcripts. Acetylation and methylation are prevalent and dynamic posttranslational modifications of transcription factors and RNA polymerases common to all three domains of life (Gu and Roeder 1997; Yu et al. 2008; Schröder et al. 2013). Given the ancestral role of these posttranscriptional modifications in modulating transcription initiation and their conserved relationship with transcript stability observed in this study, these histone modifications (and other epigenetic modifications) may have first evolved as a byproduct of transcription regulation. Supporting this notion, histone modifications occur in the direction of transcription, and H3K4me3 is specifically associated with productive elongation and maturation of stable RNA products (Sims et al. 2007), whereas H3K27ac precedes this step (Kaikkonen et al. 2013).

In summary, csRNA-seq is a simple, versatile, and highly sensitive method to profile transcription initiation and regulation from RNA alone. By yielding single-nucleotide resolution TSS location data, csRNA-seq represents an alternative to H3K27ac ChIP-seq or methods that profile open chromatin to identify active regulatory elements and could empower the annotation of GWAS risk variants with regulatory functions in different tissues.

## Methods

### Capped small RNA-seq

A comprehensive description of the method and analysis software can be found in the Supplemental Methods as well as at http://homer.ucsd.edu/homer/ngs/csRNAseq/. Small RNAs of ∼20–60 nt were size-selected from 2–15 µg of total RNA by denaturing gel electrophoresis (Supplemental Fig. S7). A 10% input sample was taken aside and the remainder enriched for 5′-capped RNAs with 3′-OH. Monophosphorylated RNAs were selectively degraded by Terminator 5′-phosphate-dependent exonuclease (Lucigen). Subsequent 5′ dephosporylation by CIP (NEB) followed by decapping with RppH (NEB) augments Cap-specific 5′ adapter ligation by T4 RNA ligase 1 (NEB). The 3′ adapter was ligated using truncated T4 RNA ligase 2 (NEB) without prior 3′ repair to select against degraded RNA fragments. Following cDNA synthesis, libraries were amplified for 11–14 cycles and sequenced SE75 on the Illumina NextSeq 500.

Sequencing reads were trimmed for 3′ adapter sequences (AGATCGGAAGAGCACACGTCT) using HOMER (“*homerTools* trim”) and aligned using HISAT2 (Kim et al. 2015) with default parameters. For mapping stats and statistics, please see Supplemental Table S1. TSS clusters were defined using HOMER's *findcsRNATSS.pl* tool that automates the following analysis steps to produce an annotated list of likely TSSs: (1) Peaks of strand-specific csRNA-seq reads found within 150 bp with a minimum read-depth of seven reads per 10^7^ aligned reads and greater than twofold reads per base pair than the surrounding 10 kb were considered for further analysis. This step eliminates loci with minimal numbers of supporting reads or regions with high levels of diffuse signal. (2) Short RNA input libraries (and/or total RNA-seq) were integrated and the appropriate enrichment thresholds for csRNA-seq reads over input or total RNA-seq libraries calculated. The optimal threshold is defined as the ratio that generates the largest difference in cumulative distributions of putative TSS regions in annotated TSS regions (i.e., true positives) relative to putative TSSs identified in downstream exons (i.e., likely false positives). This semisupervised threshold detection approach is most needed when RNA quality is low. By using this approach, we were able to successfully call TSSs from libraries generated from RNA with RIN numbers as low as two.

To estimate the likely stability of transcripts initiating from each TSS, total RNA-seq reads (sense strand) are quantified from [−100,+500] relative to the TSS. “Stable TSSs” were defined as TSS clusters containing at least two per 10^7^ RNA-seq reads within this region. Bidirectional or divergent transcription for a given TSS cluster was calculated by quantifying csRNA-seq signal on the opposite strand [−500,+100] relative to the TSS. Regions with at least two csRNA-seq reads per 10^7^ were called as “bidirectional” TSSs. TSS clusters were further annotated based on their overlaps with annotated gene regions (i.e., exons, introns, etc.), and the closest annotated gene promoters were also identified to assess their distal annotation (promoter-distal TSSs defined as >500 bp from annotated gene TSSs). TSSs from alternative transcription initiation methods were analyzed using the same pipeline as described for csRNA-seq to ensure a fair comparison among assay types. Modifications were made to adapter trimming as needed per data set to remove the correct 3′ adapter, and for assays that use paired end sequencing, only the read encoding the 5′ initiation site was used in downstream analysis.

### Total RNA-seq

Strand-specific total RNA-seq libraries from ribosomal RNA–depleted RNA were prepared using the TruSeq kit stranded total RNA library kit (Illumina) and sequenced PE100 on Illumina HiSeq 2500.

### RNA isolation and samples

*N. vectensis* (planula stage) was kindly provided by Drs. James Gahan and Fabian Rentzsch (University of Bergen) and shipped on dry ice but arrived defrosted. *N. crassa* was provided by Dr. Jason Stajich (University of California [UC], Riverside) and grown in Vogels media under constant light and gentle agitation (Wang et al. 2015). Rice was grown in the SALK greenhouse with 12-h light and leaves from adult plants provided by Dr. Joanne Chory (Salk Institute for Biological Studies). All samples were flash frozen in liquid N_2_, pulverized with a mortar and pestle, and RNA extracted using TRIzol LS as described by the manufacturer. *C. owczarzaki* RNA (Sebé-Pedrós et al. 2016) was gifted by Dr. Iñaki Ruiz-Trillo (Institut de Biologia Evolutiva; CSIC-Universitat Pompeu Fabra). Human H9 cell RNA was provided by Yuanyuan Li and Mark H. Tuszynski (UC San Diego). H9 cells were grown as previously described (Lu et al. 2017) and RNA isolated using a Qiagen RNA kit. K562 cells from Dr. Xiang-Dong Fu (UC San Diego) were grown in RPMI 1640 + L-Glutamine with heat inactivated 10% FBS (Biowest S1620, lot 61N16) and 1 × Pen/Strep (Gibco 15140-163) and 1 × L-Glutamine (Gibco 25030-164) in T75 flasks at 37°C with 5% CO_2_. HCT116 CMV-osTIR1 RAD21-mAC cells were obtained from Masato T. Kanemaki (Natsume et al. 2016) and cultured in McCoy's 5A medium supplemented with 10% FBS. Cells were washed twice in 1× cold PBS (Gibco 10010023) and RNA isolated using TRIzol LS. Murine BMDMs were isolated, cultured, and RNA extracted as previously described (Link et al. 2018).

### Integrated NGS data analysis

General NGS analysis was performed using HOMER (Heinz et al. 2010) unless stated otherwise. A complete list of used and generated data are listed in Supplemental Table S2. ChIP-seq, DNase-seq, and ATAC-seq data were aligned using HISAT2 (Kim et al. 2015) with default parameters to the appropriate genome (human: GRCh38/hg38; mouse: GRCm38/mm10; *Nematostella*: ASM20922v1; *Neurospora*: NC12; rice: IRGSP-1.0, *Capsaspora*: C_owczarzaki_V2). Gene and promoter annotations were based on the accompanying Ensembl GTF file.

Peaks were called using HOMER's *findPeaks.pl* in either “histone” (histone modifications, default parameters) or “factor” (DNase/ATAC-seq, parameters “-fragLength 50 -size 75 -minDist 75 -F 2 -L 1”) mode to identify broad or focal peaks, respectively, using ChIP input experiments as a control for both types of analysis. Identification of overlapping or specific peaks/TSS, as well as overlaps between TSS and genome annotations or ChromHMM annotations, were calculated using HOMER's *mergePeaks* command. Overlapping TSS clusters were defined by TSS clusters located within 150 bp on the same strand. Differentially regulated TSS/peaks were calculated by first merging features from each condition (or assay) into the union of nonredundant features using *mergePeaks*. Then raw read counts associated with each feature across all experiments was quantified with *annotatePeaks.pl* and significantly differentially enriched TSS/peaks (greater than twofold, <5% FDR) determined by DESeq2 (Love et al. 2014). Normalized histograms, heatmaps, and read count totals at TSS clusters or ChIP-seq peaks were calculated using HOMER's *annotatePeaks.pl* and reported relative to a total of 10^7^ uniquely aligned reads per experiment. Gene metaplots were created using HOMER's *makeMetaGeneProfile.pl*. Strand-specific transcriptomics data were reported relative to the 5′ end of sequencing reads, whereas ChIP-seq and DNase-seq were reported +75 and +35 nt relative to the 5′ end of the sequencing reads approximating the nucleosome dyad or middle of the DNase fragment, respectively. Quantification of histone modifications associated with each TSS was performed from 0 to +600 to capture the signal located just downstream from the TSS. When reporting log_2_ ratios between read counts a pseudocount of one read per 10^7^ aligned reads was added to both the numerator and denominator to avoid divide by zero errors and buffer low intensity signal. Plotting was performed using Excel and R (R Core Team 2018). DNA nucleotide frequencies relative to TSS were generated using HOMER's *annotatePeaks.pl*.

Known motif enrichment and de novo motif discovery were performed using HOMER's *findMotifsGenome.pl* using default parameters. When analyzing csRNA-seq TSS, motifs were searched from −150 to +50 relative to the primary TSS of a TSS cluster. DNase/ATAC-seq peaks and H3K27ac peaks were analyzed from −100 to +100 and −500 to +500 relative to the center of the peaks, respectively, reflecting the locations where most TF motifs are located relative to each feature. Motif enrichment heatmaps were generated by combining known motif enrichments across experiments and then clustering the logP enrichment values by correlation coefficient (Cluster 3.0) (de Hoon et al. 2004) and visualizing the resulting heatmap using Java TreeView (Saldanha 2004).

## Data access

All sequencing data generated in this study have been submitted to the NCBI Gene Expression Omnibus (GEO; https://www.ncbi.nlm.nih.gov/geo/) under accession number GSE135498. The updated HOMER software is available at http://homer.ucsd.edu/ and as Supplemental Code.

## Supplementary Material

Supplemental Material
