# Supplementary material for: Identification and dynamic quantification of regulatory elements using total RNA
Source: Genome Res. 2019 Nov;29(11):1836–46. doi: 10.1101/gr.253492.119 (PMC6836739; doi:10.1101/gr.253492.119)
Supplement: Supplemental Material [file supp_29_11_1836__index.html]

Identification and dynamic quantification of regulatory elements using total RNA — Supplemental Material 

# Identification and dynamic quantification of regulatory elements using total RNA

## Supplemental Material

- Supplemental\_Code.zip
- Supplemental\_Materials\_and\_Methods.pdf
