# Supplementary material for: Identification and dynamic quantification of regulatory elements using total RNA
Source: Genome Res. 2019 Nov;29(11):1836–46. doi: 10.1101/gr.253492.119 (PMC6836739; doi:10.1101/gr.253492.119)
Supplement: Supplemental Material [file supp_gr.253492.119_Supplemental_Code.zip › tutorial.pdf]

HOMER

Software for motif discovery and next-gen sequencing analysis

---

csRNA-seq Analysis Tutorial

Sequencing the 5' end of cap-protected RNAs enables the identification of Transcription Start Sites (TSS) at nucleotide resolution. Several varieties of this method exist, including CAGE, TSS-Seq, START-seq, PRO-Cap, GRO-cap, CAP-seq, 5'RNA-Seq, 5'GRO-Seq etc., and each are designed to collect the 5' ends of RNAPII transcripts for sequencing but use different enzymatic or enrichment strategies to achieve their goal. We recently developed [capped short] csRNA-seq to map nascent initiation sites, which identifies initiating transcripts by sequencing short (20-65nt) RNAs with 5' caps extracted from total RNA. This strategy targets RNAs created just after RNAPII has initiated, transcribed a short distance, and added a 5' cap to the nascent RNA. Like other methods that attempt to isolate nascent initiation sites like Start-seq, GRO-cap/PRO-cap/5'GRO-seq/CoPRO/etc., csRNA-seq identifies TSS from all RNAPII transcripts, including mRNAs, primary miRNA transcripts, enhancer RNAs (eRNA), promoter anti-sense transcripts, and other stable and unstable transcripts. These techniques are particularly powerful for identifying active regulatory elements (enhancers + promoters) and assessing their activity in a quantitative manner with relatively low sequencing coverage. For example, 20-40 million reads from a csRNA-seq experiment might yield the same number of reads at enhancers as 200-400 million reads in a 5'RNA-Seq experiment.

Introduction to Transcription Initiation

To understand the analysis of 5'RNA data, it is worth taking a moment highlight that there are multiple 'types' of promoters in living organisms. First of all, there are different RNA polymerases including RNA polymerase I (rRNA), II (mRNA, lncRNA, miRNA), III (tRNA), IV(plant specific), viral polymerases, etc., and each polymerase has different mechanisms directing transcription initiation that may vary between organisms. Also be aware that different RNA polymerases may generate RNAs with different modifications and may or may not be present in your sample depending on how the experiment was performed. Most researchers are interested in RNA polymerase II transcripts (i.e. mRNA, eRNA, etc.), and as a result, most methods focus on the identification of RNAs containing a 7-methylguanosine cap protecting their 5' end.

With respect to RNA polymerase II initiation sites, there are two generally recognized 'types' of TSS. Sharp (or Focused) TSS initiate transcription from a single nucleotide (or +/- 2 nt) and resemble the promoters found in molecular biology text books. They often contain well define core-promoter elements such as the TATA box and usually initiate transcription from a purine preceded by a pyrimidine (PyPu, e.g. CA, with the A being the initiating nucleotide).

The other, more common TSS is a broad (or dispersed) TSS or TSS cluster. These promoters initiate transcription from several different sites within a large area (often 50-100 nt in size). These promoters usually lack clear core promoter elements (usually lack a TATA box), but each individual initiation site still usually initiates on a purine preceded by a pyrimidine (PyPu).

The csRNA-seq Method

Capped small RNA-seq (csRNA-seq) accurately maps RNA polymerase II transcription start sites (TSSs) from any tissue or organism where RNA can be extracted. csRNA-seq is an adaptation of Start-seq (Nechaev et al., Scruggs et al.) and small RNA sequencing (Lister et al., Gu et al.). The method captures small (<60 nt), 5'-capped RNAs which associate with newly initiated RNAs, providing data similar to Start-seq or 5'GROseq/GROcap(Kruesi et al., Lam et al.). However, csRNA-seq works with total RNA, eliminating the requirement of nuclei isolation or run-on. csRNA-seq is thus widely applicable and facilitates accurate maps of TSSs of stable (i.e. mRNAs, ncRNAs) and unstable transcripts (i.e. enhancer RNAs, pre-miRNAs, divergent transcripts, antisense transcripts) from fresh, frozen or archived tissues or samples – basically any material

where quality RNA can be isolated from.

False TSS - be careful of artifacts

A quick note about artifacts in csRNA-seq (and other 5'RNA) data: Most 5' RNA-Seq methodologies work by enriching for 5' cap-protected RNA, which means that most of the sequence data describes true 5' RNA ends, but a fraction of it may be noise from random RNA fragments that may lack a 5' cap. In particular, highly expressed RNAs may yield reads along exons or highly transcribed gene bodies, giving the appearance of alternative TSS that are likely false positives. Because of this, it is recommend that a non-capped control sample or traditional RNA-Seq data set is used as a "background" when analyzing this type of data. This approach (described below) may remove several real TSS from the results, but it is also likely to remove a large number of false positives and clean up your analysis.

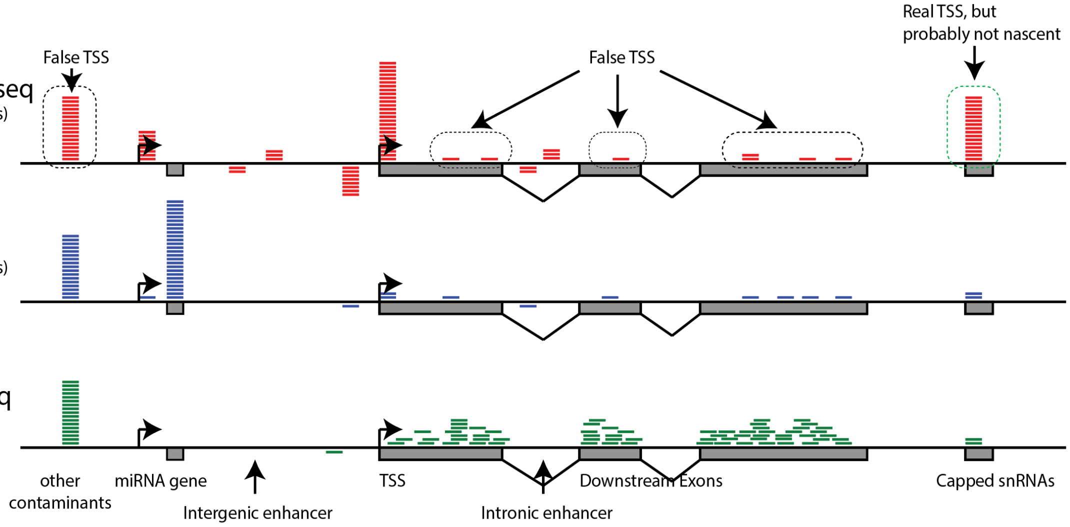

Trans-splicing of transcripts (where the 5' end of one transcript is added to the front of another) and recapping (where a transcript is cleaved and a new cap placed on the truncated product) are two phenomena you may want to think carefully about when analyzing 5' RNA data. Trans-splicing will create false negatives and recapping will create false-positives. In certain organisms, such as C. elegans, trans-splicing is very common, making csRNA-seq or 5'GRO-Seq much better assays for identifying TSS than 5'RNA-Seq (i.e. measuring the 5' RNA ends before they have a chance to trans-splice). In other organisms (e.g. mouse, human, fly, etc.) trans-splicing appears to be rare. The degree to which transcripts are 'recapped' is a matter of debate because it can be hard to distinguish noise in these assays from true recapped transcripts, true alternative TSS (in the genome). For practical purposes the analysis framework below assumes capped 5'RNAs found in the context of highly expressed exons to be likely false positives.

Analyzing csRNA-seq data (initiated transcripts)

Nearly all of this analysis can be applied to related 5'RNA sequencing methods as well, including 5'RNA-seq, 5'GRO-seq, GRO-cap, PRO-cap, CoPRO, Start-seq, TSS-seq, etc.

1. FASTQ trimming

csRNA-seq captures short RNAs that are likely shorter than the read-length used for sequencing. To properly map and analyze the data, you will need to trim the 3' adapter from the end of the sequences. The following command uses homerTools, but you can use your favorite read trimming program too.

homerTools trim -3 AGATCGGAAGAGCACACGTCT -mis 2 -minMatchLength 4 -min 20 exp1.csRNA.fastq.gz
#repeat for all samples and input controls

This command allows up to 2 mismatches when matching the barcode sequence, will only allow a

match if at least 4 nucleotides match at end of the read, and will automatically discard trimmed sequences that are less than 20 nucleotides long. The command will produce 2 files: *exp1.csRNA.fastq.gz.trimmed* (FASTQ containing trimmed sequences) and *exp1.csRNA.fastq.gz.lengths*, which contains a table in tab-delimited text format describing the read length distribution of the reads. Don't forget to do the same with control samples (i.e. input) that skip the 5' cap enrichment steps.

Quality Control Checks:

1. **Total number of reads** (e.g. did you get the expected number of reads in this experiment?)
2. **% of adapter-dimers**: Since csRNA-seq relies on adapter ligation, it is possible to generate adapter dimers, which are normally excluded from sequencing by size selection. Furthermore, reads trimmed to lengths less than ~15-20 nt are essentially unusable for downstream analysis due to their inability to be properly aligned to the genome.
3. **Read length distribution** (can check this here, or during step 3 below)

2. Align csRNA reads to the Genome

In general, RNA molecules identified by csRNA-seq should be freshly initiated without having time to be spliced or processed in any meaningful way (aside from the cap being added to the 5' end of the RNA). In that sense, it may be defensible to align reads with either a DNA (bwa, bowtie2) or RNA (STAR, Hisat2) alignment algorithm. However, we recommend aligning the reads with a splicing-aware (i.e. RNA) alignment program in case there is significant contamination from spliced RNA molecules in the sample. It is better to know they are there and potentially remove them later than to bury your head in the sand. For example, to align with STAR you might run:

> **STAR --genomeDir GenomeIndexForhg38/ --runThreadN 24 --readFilesIn exp1.csRNA.fastq.gz.trimmed --outFileNamePrefix exp1.csRNA.fastq.gz.trimmed. --outSAMstrandField intronMotif --outMultimapperOrder Random --outSAMmultNmax 1 --outFilterMultimapNmax 10000 --limitOutSAMoneReadBytes 10000000**
> #repeat for all samples and input controls

This command will align the reads to create the file *exp1.csRNA.fastq.gz.trimmed.Aligned.out.sam*, which should contain alignments for all of the csRNA-seq data. It also has options set to output a single alignment position in the case of a read mapping to multiple locations in the genome (i.e. multimapper). The *exp1.csRNA.fastq.gz.trimmed.log.final.out* file in this example should contain alignment statistics. Control samples should also be aligned in the same manner to facilitate comparisons later.

Quality Control Checks:

1. **Overall alignment %**: Ideally, this should be high (>70-90%) depending on organism and completeness of the genome assembly.
2. **Uniquely alignable vs. multimapper** %: The relative fraction of multimappers may depend biologically on the sample, organism, and cell type, and may vary technically with RNA quality (e.g. perhaps more rRNA is in the library).

3. Create HOMER Tag Directories

To leverage HOMER's quality control routines and prepare tag directories for use with HOMER's other utilities, run the makeTagDirectory command on the alignment file. This command will also analyze the sequence context of the reads, and requires the genome as well (either as a FASTA file, or if it's preconfigured in HOMER, a genome version name e.g. hg38).

> **makeTagDirectory Exp1-csRNA-tagDir/ exp1.csRNA.fastq.gz.trimmed.Aligned.out.sam -genome hg38.genome.fa -checkGC -fragLength 150**
> #repeat for all samples and input controls

This command will create a new directory (Exp1-csRNA-tagDir/) that will contain read information and several QC and metadata files that you can look at. The command also includes options that will trigger the program to check the nucleotide content of the reads and their genomic context out to 150 nt from the 5' end of the reads.

Other potential options to use:

> **-keepOne** : Normally homer discards multimappers, this option will tell it to keep the primary alignment even if the MAPQ < 10
> **-minlen <#>** and **-maxlen <#>** (Filter reads with lengths outside this range, could be useful to exclude specific contaminants, i.e. "-maxlen 65" could help avoid a stable snRNA that's 70nt long)
> **-single** : If using an organism with a genome composed of lots of contigs/scaffolds, you may be required to specify "-single" to modify how it stores reads in the directory

Quality Control Checks:

1. **Read clonality/PCR duplicates**: This information is available in the *tagCountDistribution.txt* file located in the completed tag directories. csRNA-seq data is like a hybrid between ChIP- and RNA-seq. It has positional information like you would expect from ChIP-seq, but a dynamic range like RNA-seq. I would not recommend removing duplicate reads as it would unnecessarily limit the dynamic range of the data (although use of UMIs would be best). Usually the input libraries, which often contain high levels of miRNAs, should contain more 'highly clonal' locations in the data. A typical example of good data might look like the following (plotted as a log-log plot). Although rare, look out for anything that has a mode other than 1, which might be indicative of a low complexity library.

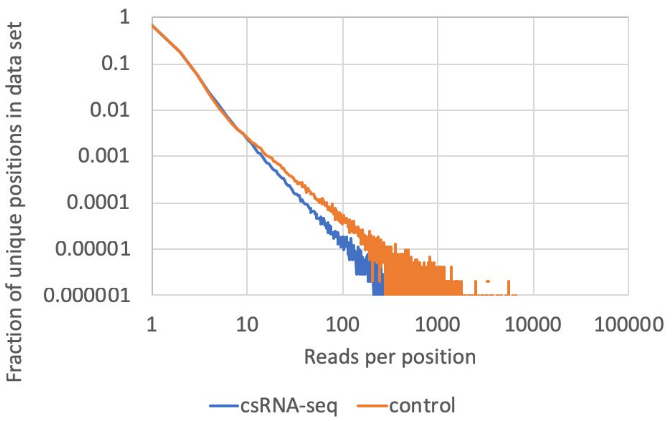

2. **Read length distribution**: The distribution of read lengths for each experiment will be available in the *tagLengthDistribution.txt* file located in the completed tag directory. Most csRNA-seq reads are between 20-55 nt in length, with relatively few longer than 60 nt. The distribution normally peaks around 30 nt or so. Depending on the source of the sample, there may be spikes in the distribution that correspond to specific high-abundance RNAs that may be present in the sample. For input controls, there is normally a spike in ~22 nt RNAs corresponding to miRNAs in the sample. Input sample may contain similar or unique spikes at specific lengths depending on the presence of high abundance RNAs or fragments of rRNA in the sample. A typical csRNA-seq library and it's control from a human cell line look like this:

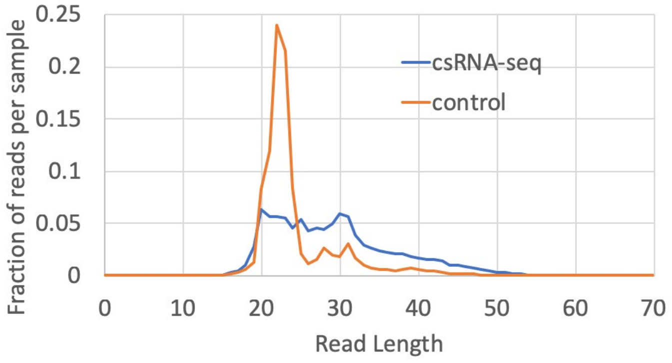

3. **Nucleotide preferences near the 5' end of the read**: [Requires a genome to be specified or FASTA file] The composite nucleotide frequency in the genome relative to the 5' end of each read is available in the *tagFreqUniq.txt* and *tagFreq.txt* files in the tag directory. The files are similar, except that the tagFreq.txt weights each position based on the total number of reads at each position, while the tagFreqUniq.txt file only counts each position in the genome once, regardless of how many reads align to each position. Normally you can ignore the tagFreq.txt file since it will be heavily influenced by any high abundance/clonal reads in the sample. *What to look for*: Most csRNA-seq data should have a strong preference for Initiator-like sequences near the 5' end (usually $C_{-1}A_0$). Most species also show evidence for a TATA box at approx. -30 bp (e.g. spike in A/T content). Most samples also have specific preferences in the region downstream of the TSS from +1 through +40. This region is normally G-rich in vertebrates. In general, Metazoans commonly display 10 nt helical fluctuations in nucleotide content downstream of the TSS (e.g. +40 to +190) likely corresponding to sequences that help rotationally position nucleosomes relative to transcription initiation. Control libraries, on the other hand, can vary widely depending on the relative fraction of non-TSS associated reads they contain. In some cases they may contain features that a good csRNA-seq library should have (i.e. as listed above), while other times they have their own sequence preferences corresponding to the types of high abundance RNAs represented in the library. Note on GC-content: Some csRNA-seq libraries are more GC-rich or less GC-rich depending on the sample being studied, which might cause the relative levels of G+C relative to A+T to change. However, good csRNA-seq libraries should still have the major features described above.

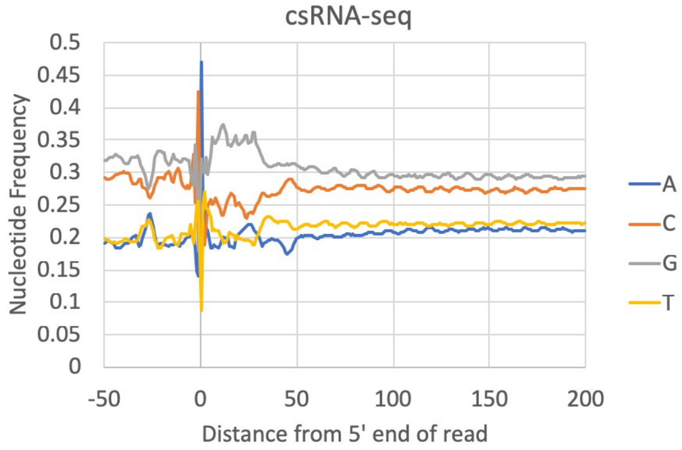

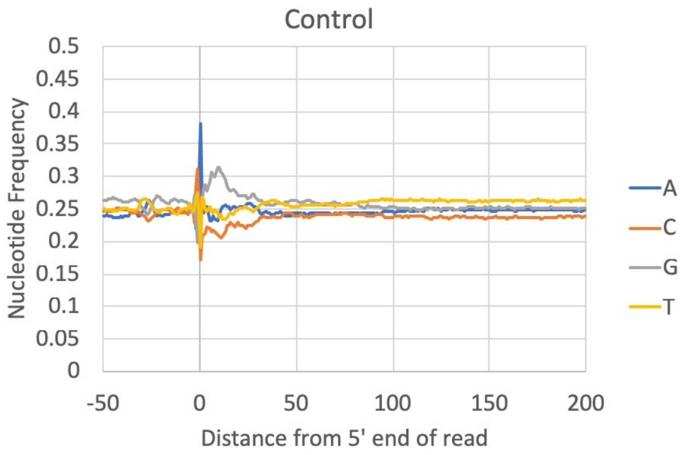

4. **Distribution of reads relative to one another**: The *tagAutocorrelation.txt* file in the tag directory contains information about how reads relate to one another in the sample. In the case of transcription initiation data, reads from TSS will typically cluster on the same strand within 50-100nt from each other. Due to the [normally] bidirectional nature of transcription from regulatory elements, reads will also typically appear upstream of TSS on the opposite strand (i.e. bidirectional transcription). Control libraries, with reads typically originating from high abundance RNAs or fragments of longer RNAs, normally don't show much enrichment for these bidirectional read clusters:

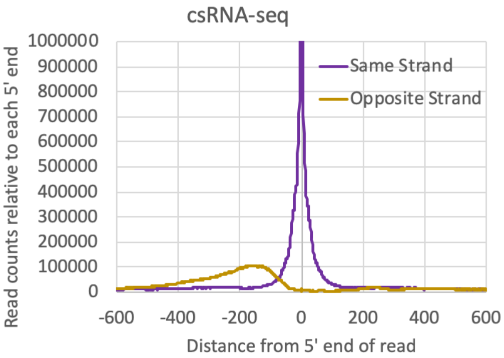

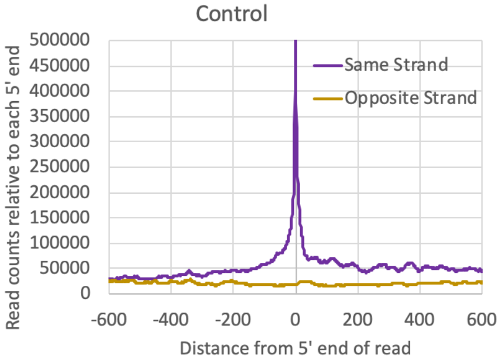

4. Create Genome Browser Visualization Files (e.g. bigWigs)

Visualizing the experiments in a genome browser is imperative to provide an independent assessment of the experiment quality and read distribution, and enables you to visualize how the experiment may relate to other data or annotations. Visualizing the experiment as the rates of transcription at single nucleotide (5' ends, i.e. sites where transcription initiates) is typically the most useful way to visualize the experiment, but you could also visualize the result more traditionally as a read-pileup showing the coverage of the reads. Note that the assay is strand specific, so separate visualization files will need to be generated for + strand and - strand

alignments. Usually, we visualize csRNA-seq data in either the UCSC Genome Browser or with IGV. In principle you can visualize the data with any genome browser. By default, these routines will normalize the resulting bedGraphs by total number of uniquely aligned reads (to an effective total of 10 million).

Creating visualization files in steps from bedGraphs:

> **makeUCSCfile Exp1-csRNA-tagDir/ -style tss -strand + > exp1.posStrand.bedGraph**
> **makeUCSCfile Exp1-csRNA-tagDir/ -style tss -strand - -neg > exp1.negStrand.bedGraph**

Note that when creating the negative strand data, it's useful to specify "-neg" to have the reads extend downward when visualized to make the separation between + and - strand reads more obvious. The above example will visualize the data at single nucleotide resolution - to visualize the total read coverage, replace "**-style tss**" with "**-style rnaseq**". The resulting bedGraph files can be gzipped and uploaded to either UCSC or used with IGV.

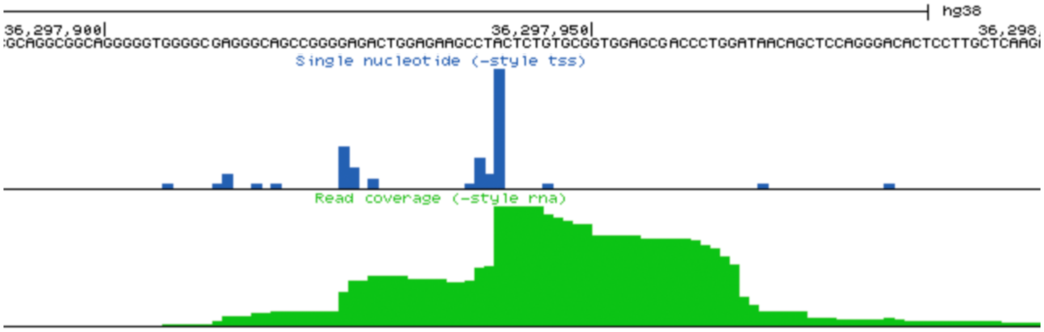

To create bigWigs from bedGraphs, you'll need UCSC's **bedGraphToBigWig** utility (more info on this stuff here), as well as a file specifying the sizes of the chromosomes:

> **bedGraphToBigWig input.bedGraph chrom.sizes output.bigWig**

Other HOMER scripts to generate visualization files:

The following scripts help automate the creation of bigWig files from HOMER-style tag directories. More information is available here. Hubs are useful in that they can be used to combine + strand and -strand bigwig files into the same container so that both strands can be very clearly visualized at the same time (like in the examples below). Both scripts support the option "-tss" and "-rna", which will trigger the creation of single nucleotide or coverage graphs, respectively.

> **makeMultiWigHub.pl <hubname> <genome> [options] -tss -d <tag directory1> [tag directory2]...**

> **makeBigWig.pl <tag directory> <genome> -tss [special options] [options]**

Quality Control Checks:

1. **General distribution of csRNA-seq reads**: You should see nice signal at the 5' end of annotated genes. Depending on the organism, you should also generally see reads initiating in the opposite direction (divergent or bidirectional transcription). In most vertebrates you should also see csRNA-seq read clusters in enhancer regions too, although the signal here will generally be lower than near most promoters. Visually inspecting areas that are thought to be super enhancers is normally a good idea as they should have evidence of enhancer transcription. Very high signal may also be present at certain snRNA, snoRNA, or other high abundance RNA.
2. **Visually check exon enrichment at highly expressed genes**: One of the main sources of contamination in csRNA-seq is from reads that map to the downstream exons from highly

expressed genes, particularly when analyzing samples with poor RNA quality (i.e. low RIN values). For example, reads from the csRNA-seq and the control sample have signal on the *ENO1* gene exons in the example below, which is highly expressed in K562 cells. Note the level of the y-axis in the upper and lower panels. This example comes from a cell line with high quality RNA, which is relatively "clean", and in this case it should be relatively easy to identify these regions as not enriched. However, the more reads that associate with these regions, the greater the risk that TSS clustering discovery will identify false positives from these regions.

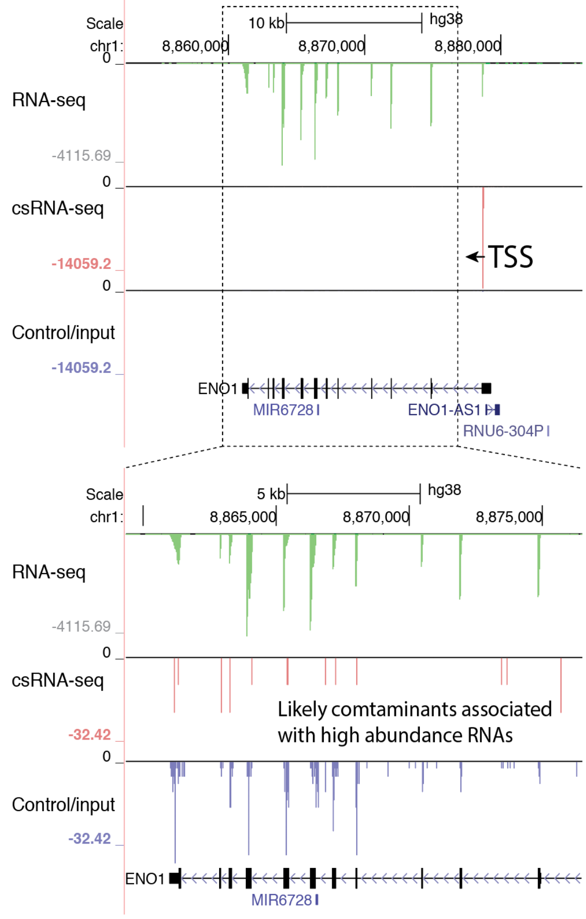

3. **Spot-check TSS and key genes**: In a vast majority of cases reads should be initiating from PyPu initiators (e.g. CA, TG, CG, TA), where the initiation site is on the Pu.

5. Finding TSS clusters from csRNA-seq data

This section will describe the identification of TSS clusters or "peaks" from csRNA-seq data. These are sites with significant initiation activity, and usually correspond to active regulatory elements in the genome. To be clear and avoid confusion, we will use the following definitions:

TSS in any individual nucleotide site that initiates transcription.
TSS cluster or **peak**: one or more TSS initiating from the same regulatory element and in the same direction (generally all TSS within the cluster are co-regulated or highly correlated). A TSS cluster is analogous to a 'peak' from ChIP/ATAC-seq experiments.

HOMER offers two related options to identify TSS clusters. If you would like to perform a quick'n'dirty analysis of the data, you can simply use **findPeaks** using the "**-style tss**" option, which does a very reasonable job of identifying TSS clusters with minimal assumptions. For a more careful and fully annotated analysis of the data, use **findcsRNATSS.pl**. The later script inherently uses the former, but also tries to use genome annotations to automatically optimize thresholds for TSS cluster detection, and will also provide various annotations that may be useful.

Simple Approach (findPeaks):

findPeaks will identify strand-specific clusters of reads in regions the size of 150 nt. It uses essentially the same approach used for ChIP-seq peak finding, except that duplicate reads are considered and the final TSS cluster will be centered on the mode (i.e. the most highly expressed individual TSS). This approach generally works well, particularly for clean samples with relatively minimal contamination from highly expressed exons.

findPeaks Exp1-csRNA-TagDir/ -i Exp1-input-TagDir/ -style tss > tssOutput.txt

This command will produce a HOMER-style peak file (similar to BED - convert with pos2bed.pl) specifying the locations of the identified TSS clusters.

Key parameters to consider:

> **-ntagThreshold <#>** : Minimum number of reads to consider before a region is considered a valid TSS cluster, measured in reads per 10 million. By default this is estimated the same way as it is for ChIP-seq by considering a Poisson distribution. This can be tricky (covered more in the findcsRNATSS.pl section), so you may want to adjust this directly to get more or less clusters.
> **-F <#>** : Fold enrichment relative input/control sample (default is 2.0). This helps remove contaminants.
> **-L <#>** : Fold enrichment relative to the surrounding 10 kb region (default is 2.0). This helps remove regions with constant signal (like downstream exons) or other odd artifacts. It may also eliminate valid TSS clusters that are 'near' other much highly expressed TSS clusters.

Full Approach (findcsRNATSS.pl):

findcsRNATSS.pl is a relatively complicated script which uses first uses findPeaks to perform an initial identification of putative TSS clusters, then attempts to annotate them and quantify different aspects of their read coverage to distinguish true TSS from likely false positives. It also provides detailed annotation information, and depending on the data that is available, will attempt to annotate TSS clusters that produce stable/unstable transcripts, uni- or bi-directional initiation, etc.

Although the program can work with simply a single Tag Directory, its full functionality requires the following input files:

- csRNA-seq Tag Directory [required] (assumed to be single end, + strand, i.e. 5' end of read represents TSS)
- input/control Tag Directory (assumed to be single end, + strand)
- RNA-seq Tag Directory (assumed to be + strand***)
- Genome FASTA file*
- GTF file*

* To use HOMER's pre-configured genome/annotations, just specify the genome version (e.g. "-genome hg38").

***** Notes about RNA-seq tag directories**

> Since different RNA-seq methods can yield different distributions of reads, you may need to create a special RNA-seq tag directory that works best for this analysis. In fact, it is best to create the tag directory such that only the 5' reads associated with each RNA-seq fragment are considered to make it a more 'fair' control for csRNA-seq. If using a truseq-style RNA-seq method

(RNA encoded on the negative strand, dUTP methods), use "-flip" (single end data) or "-read2" (paired end data) when creating the tag directory with **makeTagDirectory** for the RNA-seq. If the sample encodes the + strand, then consider using "-read1" if the sample is paired-end.

SE, +strand encoding: **makeTagDirectory TagDir/ alignment.sam**
PE, +strand encoding: **makeTagDirectory TagDir/ alignment.sam -read1**
SE, -strand encoding(i.e. TruSeq): **makeTagDirectory TagDir/ alignment.sam -flip**
PE, -strand encoding(i.e. TruSeq): **makeTagDirectory TagDir/ alignment.sam -read2**

To find TSS clusters [or peaks] run the following command (the csRNA tag directory is the first argument):

findcsRNATSS.pl <Tag Directory> [options]

findcsRNATSS.pl Exp1-csRNA-TagDir/ -o outputPrefix -i Exp1-input-TagDir/ -rna Exp1-totalRNAseq-TagDir -gtf genes.gtf -genome genome.fasta

This command will create several output files with the prefix provided with parameter "**-o <prefix>**" (default is "out"). The main output file will be *prefix.tss.txt*, which is a tab-delimited text file with each row corresponding to a TSS cluster identified in the data. The positions are given as a chromosome, start and end position, and a strand. The positions are centered on the mode of the cluster (i.e. the most prominent TSS). The other output files are mainly for QC purposes, but include:

- *out.tss.txt* : primary output file, contains positions of TSS clusters and annotation information
- *out.stats.txt* : contains general information about the run, the command used, parameters selected for the analysis.
- *out.tss.txt.freq.tsv* : Nucleotide frequencies relative to the primary TSS of each valid TSS cluster.
- *out.alltss.txt* : All putative TSS clusters, not just the valid ones, in case you want to go back and look through the others.
- *out.inputDistribution.txt* : if you used an input/control sample, this will show the CDFs for the threshold optimization.
- *out.rnaDistribution.txt* : same as the input, but for an RNA-seq control.

Key parameters to consider:

- **-ntagThreshold <#>** : Minimum number of reads to consider before a region is considered a valid TSS cluster, measured in reads per 10 million (default 7.0). Unlike peak finding for ChIP-seq, which has a high amount of background from non-specific DNA, csRNA-seq (and other RNA-based methods) can be much more sensitive, such that even single reads have a reasonable chance of representing true sites of transcription initiation. This difference is also problematic across genome sizes, where the size of the genome may not scale with the total number of TSS. The default is set to ensure TSS clusters have a reasonable number of reads to ensure their potential enrichment over control samples. You may need to adjust this lower (if the samples are really clean) or higher (if the samples are too dirty and the input/RNA-seq control data is not filtering false positive TSS clusters aggressively enough).
- **-includeSingleExons** : As explained below, the method is a little more robust/sensitive when calculating detection thresholds when using multiexon genes (single exon genes are sometimes high abundance transcripts or non-RNAPII templated transcripts). However, some organisms are comprised of mainly single exon genes (i.e. no splicing) and specifying this option will include them in the threshold defining calculations.

Working with poorly annotated genomes:

Some organisms may lack a quality set of gene annotations (i.e. no GTF file). In these cases, your options are (1) Use the simple findPeaks method above, or (2) if RNA-seq data is available, identify transcripts de novo using Cufflinks/Stringtie/etc. and use the resulting GTF file with findcsRNATSS.pl.

Steps performed by findcsRNATSS.pl to identify valid TSS clusters:

1. First, all potential TSS clusters are identified by using findPeaks to scan for regions with a high density of reads. This step is performed without any filtering for input or RNA-seq coverage to identify the locations where csRNA-seq reads are being aligned.

2. Using the GTF file (or homer annotation), the putative TSS clusters are annotated based on whether they are near an annotated TSS (within 200 bp), or if they fall on an annotated exon. The exons are further segregated based on whether they are in single exon genes, if they are part of the first exon or a downstream exon, etc. By default, HOMER will not include TSS clusters from single exon genes when optimizing detection thresholds, however, this can be overridden by specifying **-includeSingleExons** (useful for lower organisms with few spliced transcripts).

3. The reads in putative TSS clusters at annotated TSS and downstream exons are they calculated for csRNA-seq, control, and RNA-seq samples. For csRNA-seq and input, the read density corresponding to the cluster is calculated by the number of 5' read ends found with 75 bp from the primary TSS (i.e. peak size 150 centered on the TSS). For RNA-seq, reads are counted from the regions -150 to -10 relative to the TSS (i.e. upstream). By avoiding counting RNA-seq reads found at the TSS or downstream, we hope to identify TSS found within the middle of transcripts where false positives are common. The RNA-seq levels at the TSS or downstream may be expected at valid TSS depend on other criterion such as transcript stability, resulting in the erroneous discarding of valid TSS if we count the signal in these regions.

4. Since the presence of high abundance RNAs (e.g. snRNAs, snoRNAs, miRNAs, rRNA) and the quality of the RNA (i.e. RIN number) can vary between samples, cell types, tissues, organisms, etc., it is difficult to know a priori what the relative levels of input or RNA-seq should be relative to csRNA-seq levels at valid TSS. To guide this decision, the log2 ratios of csRNA-seq to both input and RNA-seq are calculated for each TSS and exon putative TSS cluster, and the log2 ratio that provides the greatest trade-off between positive identification of annotated TSS-associated clusters and exon-associated clusters is selected. For example, the graph below is created from the *prefix.inputDistribution.txt* file that is created by the script. Here, the log2 threshold is continuously lowered, and the total fraction of annotated TSS and annotated exon associated TSS are shown. The threshold is selected where the distributions are maximal (In this case at -0.34). You can specify "-fisherExact" to instead select the log2 ratio where the hypergeometric enrichment is max (takes longer to compute). For comparison, a sample with significant exon contamination but low read coverage in the input is shown next to it.

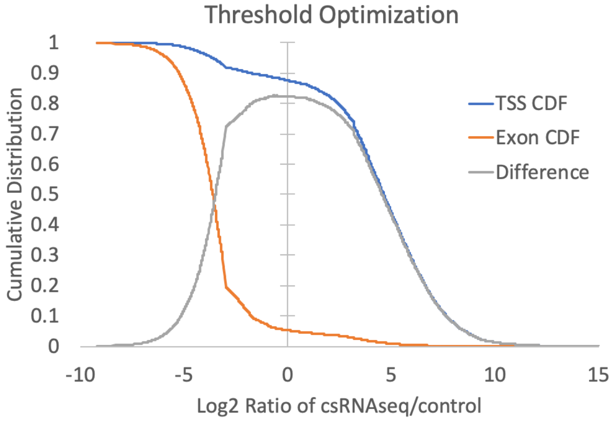

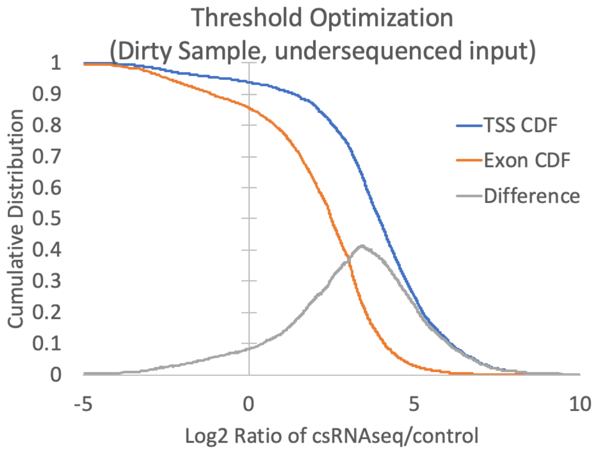

5. Once optimized thresholds are determined, they are then applied equally to *ALL* putative TSS clusters (regardless of annotation) to establish a set of valid TSS clusters. If both control/input and RNA-seq tag directories are provided, each threshold will be applied independently (i.e. valid TSS cluster must pass both thresholds).

Annotation performed by findcsRNATSS.pl:

- **TSS cluster shape** (aka focusRatio) : Ratio of reads found near the primary TSS [-5,+5] relative to the full surrounding region [-75,+75]. High focus ratios (approaching 1.0) indicate that all of the reads originate from single location within the promoter, making it a *sharp/focused* TSS cluster. Lower ratios indicate that the region has multiple strong TSS diffused over a larger region, making it a *broad/dispersed* TSS cluster. This value is also calculated by findPeaks when using the '-style tss' option.
- **General annotation** : Type of TSS cluster and the Gene Identifier associated with it, which can include:
  - tss : likely annotated transcript/gene TSS
  - tssAntisense : antisense to an annotated TSS
  - singleExon : TSS for a single exon transcript/snRNA/snoRNA/etc.
  - firstExon : Found downstream of the annotated TSS in the first exon
  - otherExon : TSS found in a downstream transcript, sense direction
  - otherExonBidirectional : Found in downstream exon, but there is also evidence for bidirectional transcription increasing the likelihood that it may be a valid TSS.
  - other : everything else (enhancers, etc.)
- **Closest annotated TSS** : including the distance, gene type, and gene name (for linking for regulatory purposes)
- **Promoter proximal vs. distal** : relative to annotated promoters, default definition is >500bp for a distal TSS cluster)
- **Stable vs. Unstable Transcript** : If RNA-seq is available, TSS clusters will be

annotated based on whether stable RNA is detected downstream [-100, +500]. By default, if at least 2 reads (per 10 million) are found, the transcript is considered stable.

- **Bidirectional vs. unidirectional** : If csRNA-seq reads (2 per 10 million) are found in the antisense direction [-500,+100] relative to the primary TSS, the cluster is considered bidirectional.
- **Regulatory Element Configuration**: Combines the stable/unstable and bidirectional annotations into a 1/2-letter code:
    - SS (TSS cluster and antisense direction produce stable transcripts)
    - SU (TSS cluster is stable, antisense direction is unstable)
    - S (TSS cluster is stable and unidirectional)
    - US (TSS cluster produces an unstable transcript, but the antisense direction creates a stable one)
    - UU (TSS cluster produces two unstable transcripts in sense and antisense directions)
    - U (TSS cluster is unstable and unidirectional)

Quality Control Checks:

1. **Total number of TSS clusters and their distribution**: Data generated from mammalian cell lines will typically have 30-100k+ TSS clusters, and tissues a little less than that. Numbers that vary greatly above or below that number might require a closer look. Other species can be variable.
2. **Spot check TSS clusters in browser** : Informaticians don't like this as much, but it is important to check examples of the TSS clusters called with a genome browser to see if the identified sites look "normal". This works best if other data can be viewed at the same time (i.e. load tracks for RNA-seq or any other experiments you may have). For example, are the TSS clusters located near the ends of transcripts?
3. **Nucleotide Frequencies at the primary TSS** : A quick quality control check provided by findcsRNATSS.pl is a file containing the nucleotide frequencies found near the primary TSS from each cluster (prefix.tss.txt.freq.tsv). You can also calculate these for any set (or subset) of TSS as described in the sections below. In general, mammalian TSS should resemble the human figure below, although the overall GC content may vary depending on the source of the sample. Fly was included to provide some reference of how things may vary as you investigate other metazoan TSS:

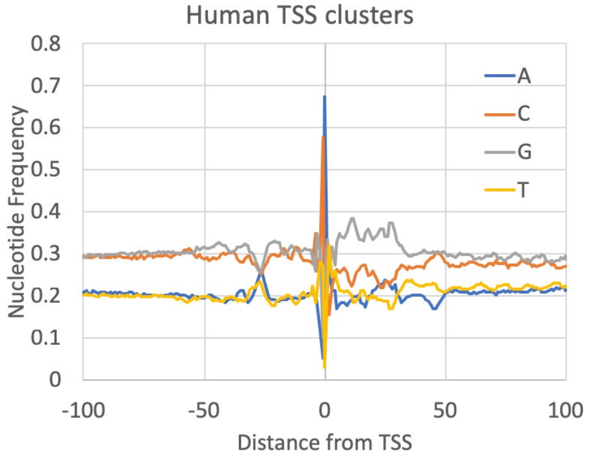

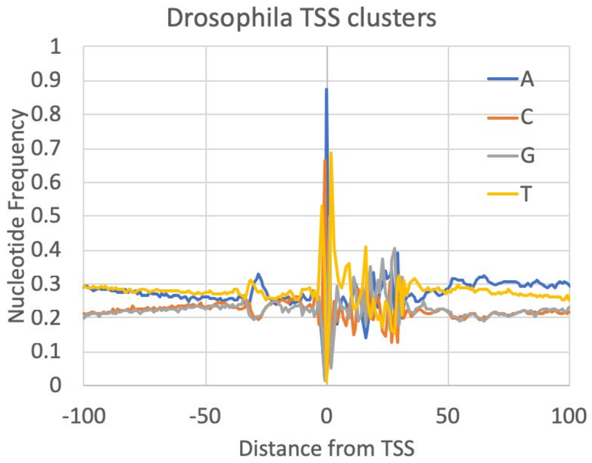

4. **Cross-reference with other data** : The best way verify the TSS clusters is to look at the TSS calls with respect to other data that provide independent evidence of transcription initiation activity. Are the TSS being called near the 5' ends of transcripts or regions with RNA-seq signal? Histone modifications like H3K4me3/2/1 and H3K27ac (histone acetylation in general) are highly associated with active initiation. TSS are also very often associated with open, nucleosome-free chromatin, meaning most sites should also overlap well with ATAC-seq or DNase-seq peaks.

The sections below cover various downstream analysis strategies that may be useful. Although this page describes how to perform these analyses with HOMER, the analysis methodology can be applied with a variety of other NGS tools as well:

6. Comparing TSS positions

To compare TSS clusters between two or more experiments, you can use HOMER's **mergePeaks** command (general page). This comparison is performed on the positions of the TSS, and does not consider quantitative changes - for that, see the next section.

To merge the TSS cluster positions from two separate experiments into a single set of non-redundant TSS clusters, use the following:

mergePeaks exp1.tss.txt exp2.tss.txt -strand > merged.tss.txt

By default, this will merge TSS clusters that overlap by at least 1 bp. It is critical to include the '**-strand**' option to ensure the TSS clusters that are merged are from the same strand. To control the distance between clusters to be merged, you can use the "**-d <#>**" to specify maximum distance between cluster centers that are merged. You can also merge multiple (3+) files at the same time. If you wish to separate out the TSS clusters that are specific to one or more of the files (instead of a single merged file), specify "**-venn <filename>**" and separate files will be produced.

NOTE: TSS clusters that are merged between two experiments will be replaced with a single region that may no longer be centered on the TSS mode (i.e. primary TSS) if the mode differs between the experiments compared (i.e. by default, merging two peaks will replace the original peaks with a single peak that is the average of their positions). If it is important that the merged peaks still be centered on the strongest TSS for subsequent analysis, you can recenter peaks on the primary TSS (i.e. the mode) for a given experiment by running the following:

> **getPeakTags exp1.tss.txt Exp1-csRNA-tagDir/ -center -fragLength 1 -strand + -start -150 -end 150 > recentered.txt**

For some analyses you may want to determine which TSS overlap a given feature without merging the features together. mergePeaks has a "-cobound <#>" feature which will use the *first file provided* and compare it to the others. For example, lets say you want to overlap TSS positions with ATAC-seq peaks from another experiment to see which ones overlap and which ones don't:

mergePeaks exp1.tss.txt atac.peaks.txt -cobound 1

This command will produce two files - *coBoundBy0.txt* (non-overlapping TSS) and *coBoundBy1.txt* (TSS that overlap with ATAC peaks). In this case, since the ATAC-seq peaks are not strand specific, we omit the "-strand" option. The number (1) associated with "-cobound 1" is not too important here and can be set to 1 - more information on mergePeaks can be found here.

Quantifying shifts in TSS positions between expeirments

To compare the positions of the primary TSS between two experiments, you can run the following command. For each TSS in the first file given to the program, it will find the closest TSS in the second file on the same strand and provide the distance (The "**-pdist2**" and "**-strand +**" are analysis/reporting options needed to tell the program to only look for peaks on the same strand and to report the relative distance):

annotatePeaks.pl <tss/peak/BED file> <genome> -p <tss/peak/BED file2> -pdist2 -strand + > outputFile.txt
annotatePeaks.pl exp1.tss.txt hg38 *-p exp2.tss.txt -pdist2 -strand* + > output.txt

Alternatively, you could also generate a histogram to see the relative distribution of TSSs from the 2nd file relative to the first (The "**-size <#>**" and "**-hist <#>**" control the region plotted and the size of the histogram bins, respectively):

annotatePeaks.pl <tss/peak/BED file> <genome> -p <tss/peak/BED file2> -size <#> -hist <#> -strand + > outputFile.txt
annotatePeaks.pl exp1.tss.txt hg38 -p exp2.tss.txt -size 1000 -hist 10 -strand + > outputFile.txt

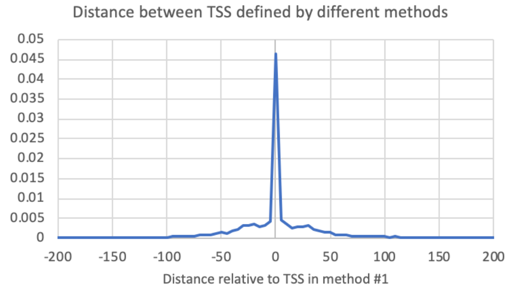

7. Quantifying TSS cluster strength and calculating differential expression

csRNA-seq is a lot like ChIP/RNA combination experiment. The signal is generally confined to well defined 'TSS/peak' regions in the genome like a ChIP-seq experiment for a transcription factor, but the dynamic range of the signal is much greater, similar to RNA-seq. In general, quantitative analysis of csRNA-seq data can be handled in much the same way as any ChIP/ATAC/RNA-seq experiment. The steps below highlight how to quantitatively compare csRNA-seq signal at different sites within the HOMER framework, although this can be done with other NGS tools in a similar manner too.

The general strategy to compare two experiments (or replicates) is the same for virtually any quantitative NGS experiment:

1. Identify the genomic features we want to compare across all experiments (i.e. merge TSS clusters across all experiments)
2. Quantify the reads for all features across all experiments
3. If replicates are available, perform differential enrichment calculations

This workflow can also apply to cases where we have 3+ experimental conditions - just replace the

final differential expression calculations with a fancier analysis, such as clustering, ANOVA, etc.

Step1: Merge TSS clusters into a single non-redundant set to quantify reads with.

> **mergePeaks exp1.tss.txt exp2.tss.txt -strand > merged.tss.txt**

If you have multiple replicates, you can either merge all of the TSS clusters identified from each individual replicate, or you could combine replicates into "meta-experiments" and perform the TSS cluster calling on the merged experiments. The latter is probably a little more sensitive, but in the end the results are *probably* not that different.

Step2: Quantify csRNA-seq read counts across experiments

> **annotatePeaks.pl merged.tss.txt hg38 -strand + -fragLength 1 *-raw* -d Exp1-tagDir/ Exp2-tagDir/ > counts.txt**

Using HOMER's annotatePeaks.pl tool, we can create a table of read counts for all TSS clusters across all experiments. When using csRNA-seq, be sure to specify "**-strand + -fragLength 1**" to ensure that the program quantifies the reads based on their 5' ends (initiation sites). You can also have the program normalize the read counts in different ways, which can be useful:

- **-raw** : Straight read counts, useful if feeding the table into another tool like DESeq2 (see below)
- **-rlog** : If R (with DESeq2) is configured on the system, this will use the rlog to transform the data - very useful if you want to do clustering/PCA/etc. as a downstream analysis. The program also supports "-vst" to use that transform instead.
- If neither is used, it will normalize the reads relative to 10 million total mapped reads per experiment.

In general, **annotatePeaks.pl** provides many useful capabilities to assist with analysis - more info here and here.

Step3: Calculate significant changes in csRNA-seq read counts (requires replicates, R/DESeq2 to be installed)

> #lets say you have 2 experiments with 2 replicates each:
>
> **mergePeaks exp1-rep1.tss.txt exp1-rep2.tss.txt exp2-rep1.tss.txt exp2-rep2.tss.txt -strand > merged.tss.txt**
>
> **annotatePeaks.pl merged.tss.txt hg38 -strand + -fragLength 1 -raw -d Exp1-rep1-tagDir/ Exp1-rep2-tagDir/ Exp2-rep1-tagDir/ Exp2-rep2-tagDir/ > rawcounts.txt**
>
> **getDiffExpression.pl rawcounts.txt exp1 exp1 exp2 exp2 > output.txt**

More information on differential analysis with HOMER can be found here.

8. Analysis of Sequence Features and Motif Discovery at TSS

Examining the nucleotide frequencies relative to TSS can reveal several interesting features, including evidence for core promoter elements, CpG islands, nucleosome positioning sequences, and other strong sequence biases that may be important for gene regulation. To find the **DNA nucleotide frequencies** relative to a set of TSS, run the following command:

> **annotatePeaks.pl <tss/peak/BED file> <genome> -size <#> -hist 1 -di > output.txt**
>
> **annotatePeaks.pl exp1.tss.txt hg38 -size 1000 -hist 1 -di > output.txt**

The first argument is the TSS cluster positions file (i.e. out.tss.txt), the 2nd argument is the genome (for custom genomes use a FASTA file). The "**-size <#>**" option specifies how large of a region you want to look for nucleotide frequencies, and the "**-hist <#>**" specifies the bin size used in the

histogram (1=single nucleotide percision). The "**-di**" option is required to tell the program to look at the DNA sequence (short of dinucleotide frequencies). For example, below is an example of nucleotide frequencies near zebrafish promoters:

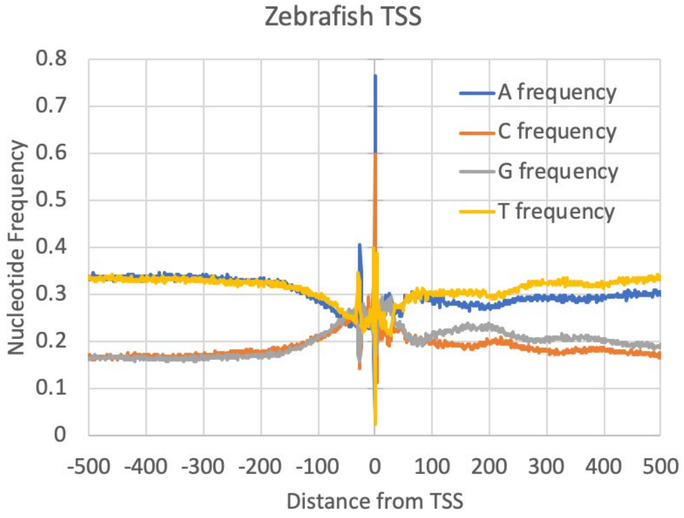

To find **DNA motifs** that are enriched near TSS clusters, you can run the following command:

findMotifsGenome.pl <tss/peak/BED file> <genome> <outputDirectory> [options]

findMotifsGenome.pl exp1.tss.txt hg38 OutputDirectory/ -size -150,50

The first argument is the TSS cluster positions file (i.e. out.tss.txt), the 2nd argument is the genome (for custom genomes use a FASTA file), and the 3rd argument is an output directory where the result files will be placed. For TSS, most of the key transcription factor motifs can *usually* be found just upstream of the TSS, unlike ChIP-seq or ATAC-seq, where the motifs are likely right in the center of the peak. Because of this, you can usually get higher sensitivity if you tell the program to modify the search to the region from -150 to +50 relative to the TSS ("**-size -150,50**", as in the example above).

Once you've identified motifs, you can check for their distribution relative to the TSS to look for additional patterns. To do this, you'll need to select one of the *.motif files identified in the motif analysis (or grab one or more from the homer/motifs/ directory). You can then run annotatePeaks.pl to create a histogram of their locations relative to the primary TSS. The "-size <#>" and "-hist <#>" specify the properties of the histogram:

annotatePeaks.pl exp1.tss.txt hg38 <u>-size 500 -hist 1 -m sp1.motif</u> > output.txt

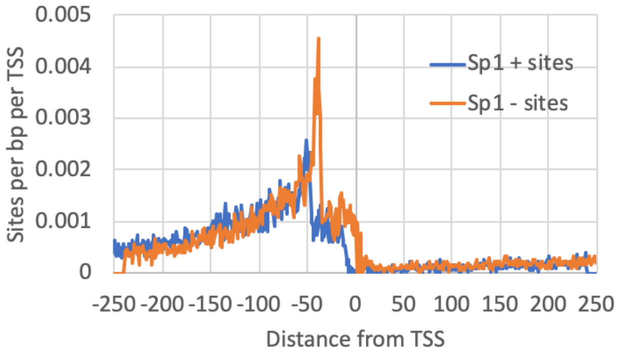

The graph above looks for where motifs are found relative to the TSS. However, another strategy is to look at where the csRNA-seq reads fall relative to the location of motifs. To do this, we first recenter our TSS/peaks/regions on the motif, and then quantify the csRNA-seq data in the surrounding regions. When centering the motif, all of the resulting peaks will have the motif site (e.g. Sp1) starting at 0 (center of the peak). When quantifying csRNA-seq reads relative to Sp1, the option "**-histNorm <#>**" triggers the program to normalize the read profile for each peak before

adding it to the composite profile (this way very highly expressed regions or other 'spikes' in the data don't drown out the other regions). The value for "**-histNorm <#>**" represents the minimum total normalized number of reads used in the normalization (this way regions with only 1 read do not create spikes in the profile either).

#first re-center the TSS on the motifs
annotatePeaks.pl exp1.tss.txt hg38 -size 500 -center sp1.motif > recentered.peaks.txt

#Then quantify reads near these peaks
annotatePeaks.pl recentered.peaks.txt hg38 -size 500 -hist 1 -histNorm 20 -d Exp1-csRNA-tagDir/ > output.txt

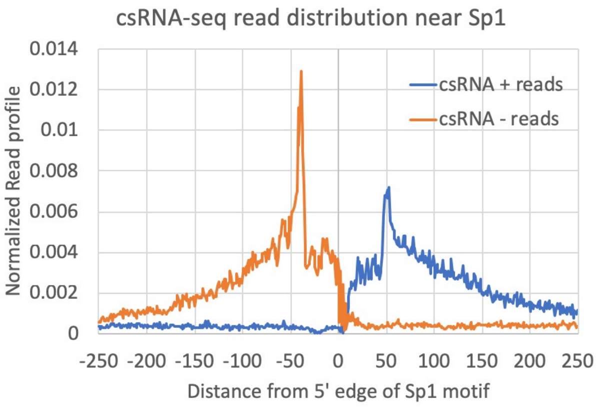

9. Comparing csRNA-seq/TSS to other data.

Integrating different types of data with csRNA-seq is similar to any integration workflow with NGS data. For example, if you have several HOMER tag directories for different types of experiments, such as histone modifications, ATAC-seq, etc., you can quantify each at TSS clusters or create histograms that show the relative enrichment of each experiment spatially.

For example, if you have ChIP-seq for H3K4me3/H3K27ac and ATAC-seq data, first create Tag Directories, and then you can map their distributions relative to the TSS clusters found from csRNA-seq (note: the "-tbp 3" option limits the number of reads (i.e. PCR duplicates) per position to 3, which removes random spikes from mapping artifacts):

annotatePeaks.pl exp1.tss.txt hg38 -size 2000 -hist 10 -tbp 3 -d ATACseq-TagDir/ H3K4me3-TagDir/ H3K27ac-TagDir/ > output.txt

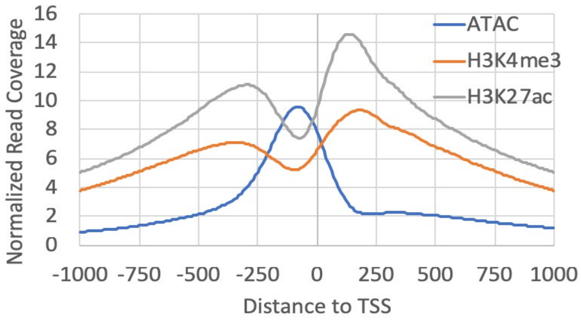

Instead of making a histogram, you could quantify the levels of each experiment within 1kb of the TSS and compare their levels at each site:

annotatePeaks.pl exp1.tss.txt hg38 -size 1000 -log -d ATACseq-TagDir/ H3K4me3-TagDir/ H3K27ac-TagDir/ > output.txt

Graphing the levels of levels of H3K4me3 vs. H3K27ac at each peak yields the following, which shows groups of TSS corresponding to H3K4me3+/H3K27ac+ gene promoter TSS and H3K4me3-/H3K27ac+ enhancer TSS:

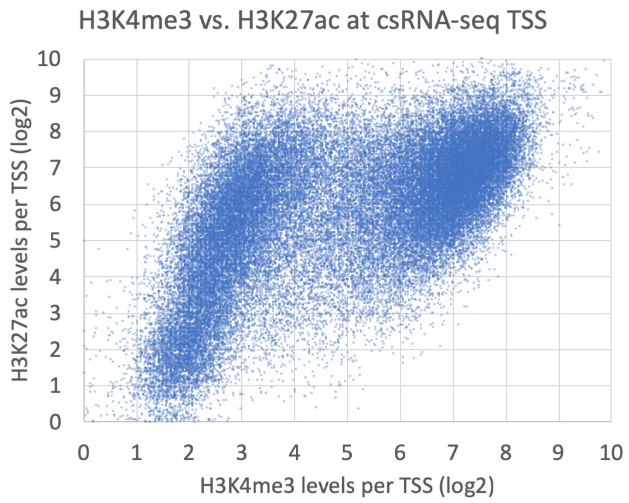

These are meant as examples - there are countless ways to analyze and compare these data!

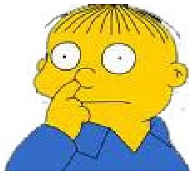

Can't figure something out? Questions, comments, concerns, or other feedback:
cbenner@ucsd.edu
