# Supplementary material for: Identification and dynamic quantification of regulatory elements using total RNA
Source: Genome Res. 2019 Nov;29(11):1836–46. doi: 10.1101/gr.253492.119 (PMC6836739; doi:10.1101/gr.253492.119)
Supplement: Supplemental Material [file supp_gr.253492.119_Supplemental_Materials_and_Methods_.pdf]

Supplementary Materials and Methods

Identification and dynamic quantification of regulatory elements using total RNA

Sascha H. Duttke*, Max W. Chang, Sven Heinz and Christopher Benner*

Sascha H. Duttke, PhD (sduttke@ucsd.edu)

Christopher Benner, PhD (cbenner@ucsd.edu)

Department of Medicine

University of California, San Diego

9500 Gilman Drive

La Jolla, CA 92093

voice: 858-534-9449

Table of contents

Supplementary Figures ………...1-9

Supplementary Methods ……………………………………………………..10-18

Supplementary Tables ………………………………………………………. 19-23

Supplementary References ………………………………………………….24-26

Supplementary Figures

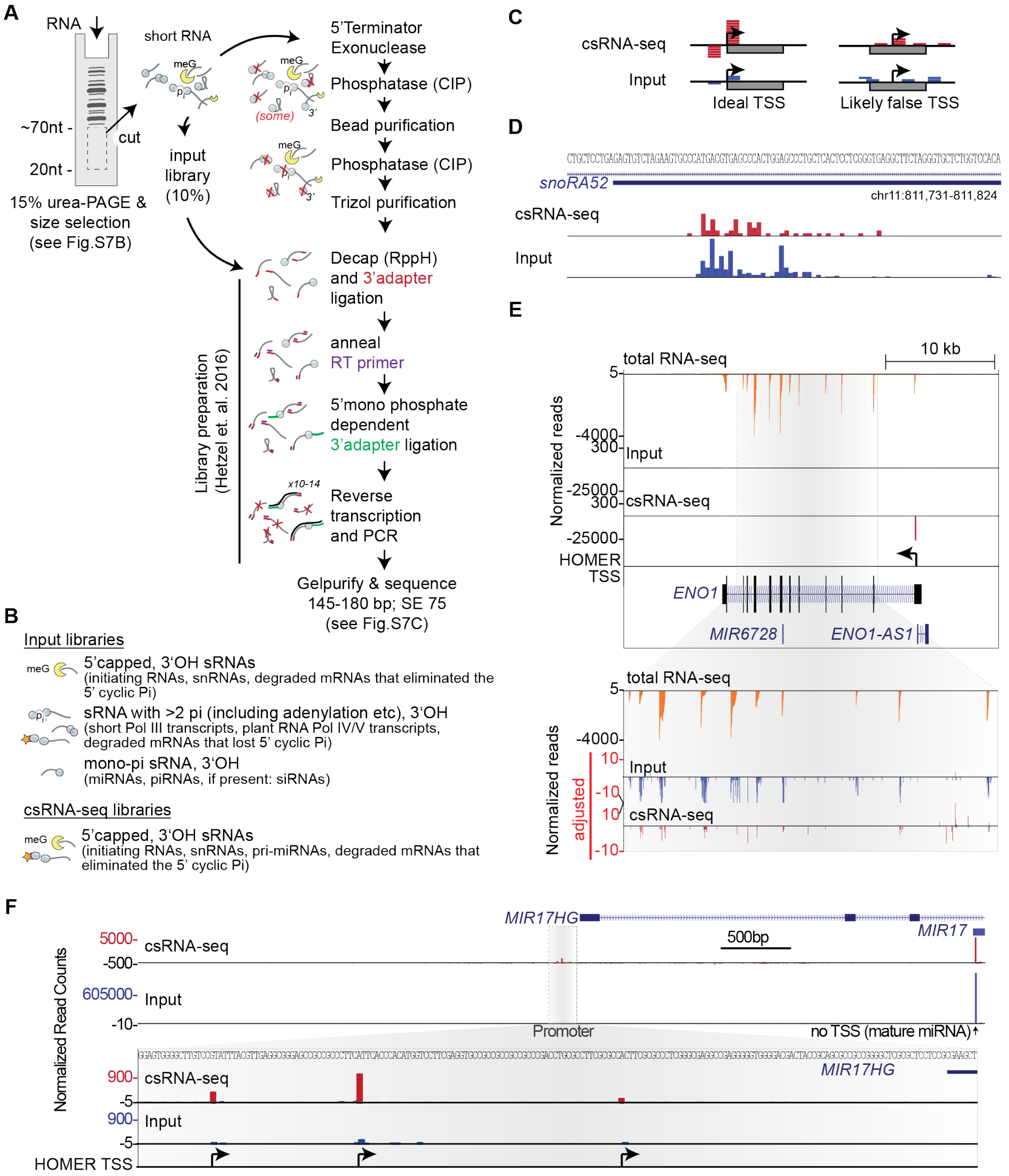

Supplemental Fig 1. Detailed overview of csRNA-seq. *(A)* Schematic protocol overview of csRNA-seq. *(B)* Summary of the major RNA species captured in input and csRNA-seq libraries. *(C)* Example outline of an ideal and likely false TSS due to sequence tags from degraded RNA. **Supplemental Fig 1 (continued).** *(D)* Example locus of *snoRA52* with reads that likely do not present a real TSS. *(E)* Example locus of the *ENO1* gene with exonic reads that likely do not present real TSS. Note: samples were scaled for better presentation. *(F)* Example locus of a pri-miRNA, captured mature miRNA and called TSS on the *MIR17* locus. Note the different scale for csRNA-seq and input libraries.

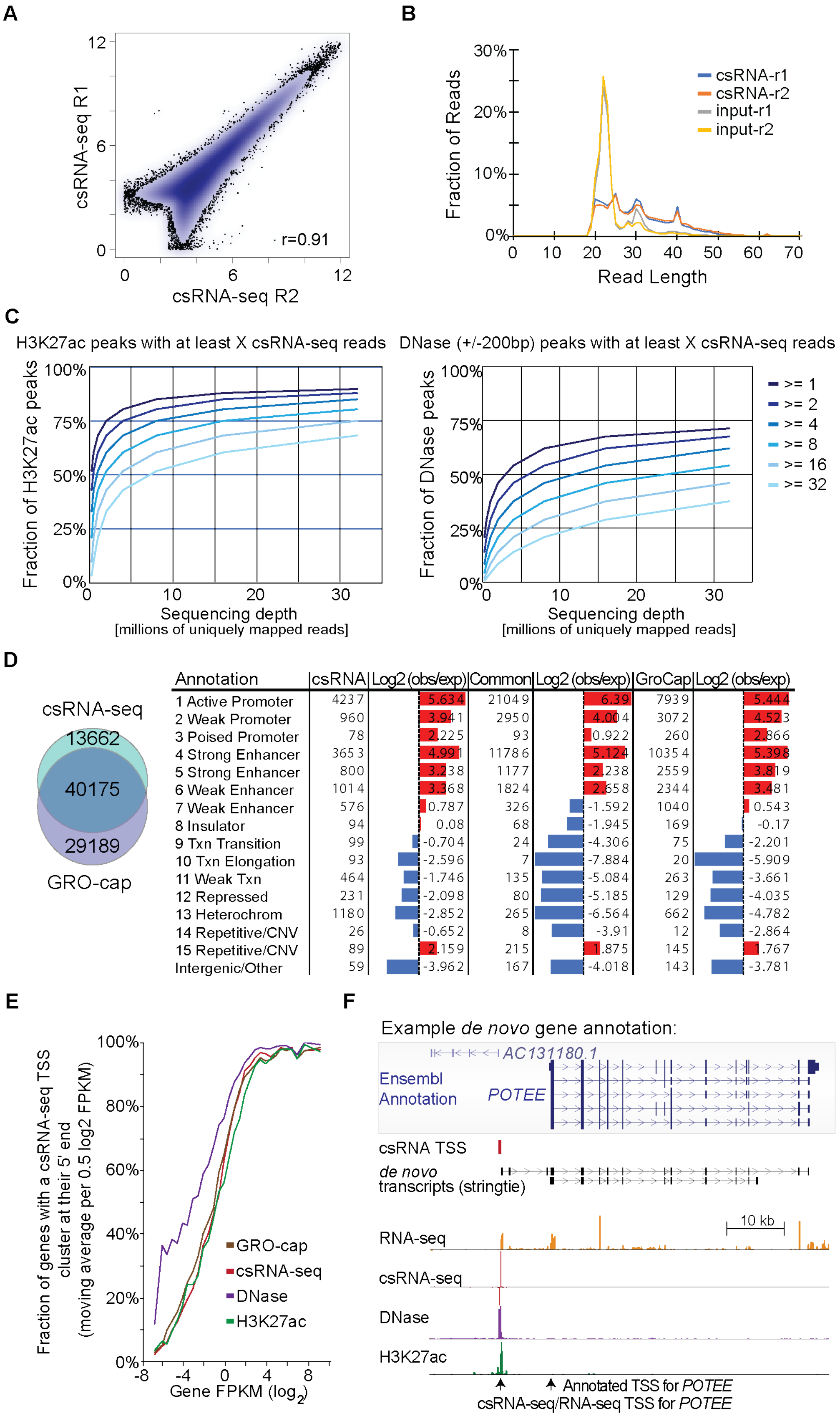

Supplemental Fig 2. Attributes of csRNA-seq data analysis

Supplemental Fig 2 (continued). *(A)* Scatter plot comparing csRNA-seq read density at TSS clusters of csRNA-seq replicates from two independent K562 cell cultures. *(B)* RNA length comparison of csRNA-seq and input libraries on K562 cells. *(C)* Plot of sequencing depth vs. sensitivity for csRNA-seq libraries investigated by saturation analysis vs. H3K27ac ChIP-seq and DNaseI-seq data in the human genome. Fewer reads are required for smaller genomes. *(D)* Comparison of csRNA-seq and GRO-cap (Core et al. 2014) TSS clusters identified in K562 cells and their enrichment in chromatin states defined by ChromHMM. *(E)* Sensitivity analysis showing the fraction of gene loci with csRNA-seq TSS clusters found at the 5' end of gene mRNA transcripts as a function of gene transcript expression in K562 cells. The analysis was also repeated for GRO-cap TSS, H3K27ac peaks, and DNase hypersensitive regions. *(F)* Example of gene locus (*POTEE*) with novel gene TSS defined by csRNA-seq and *de novo* assembly of RNA-seq.

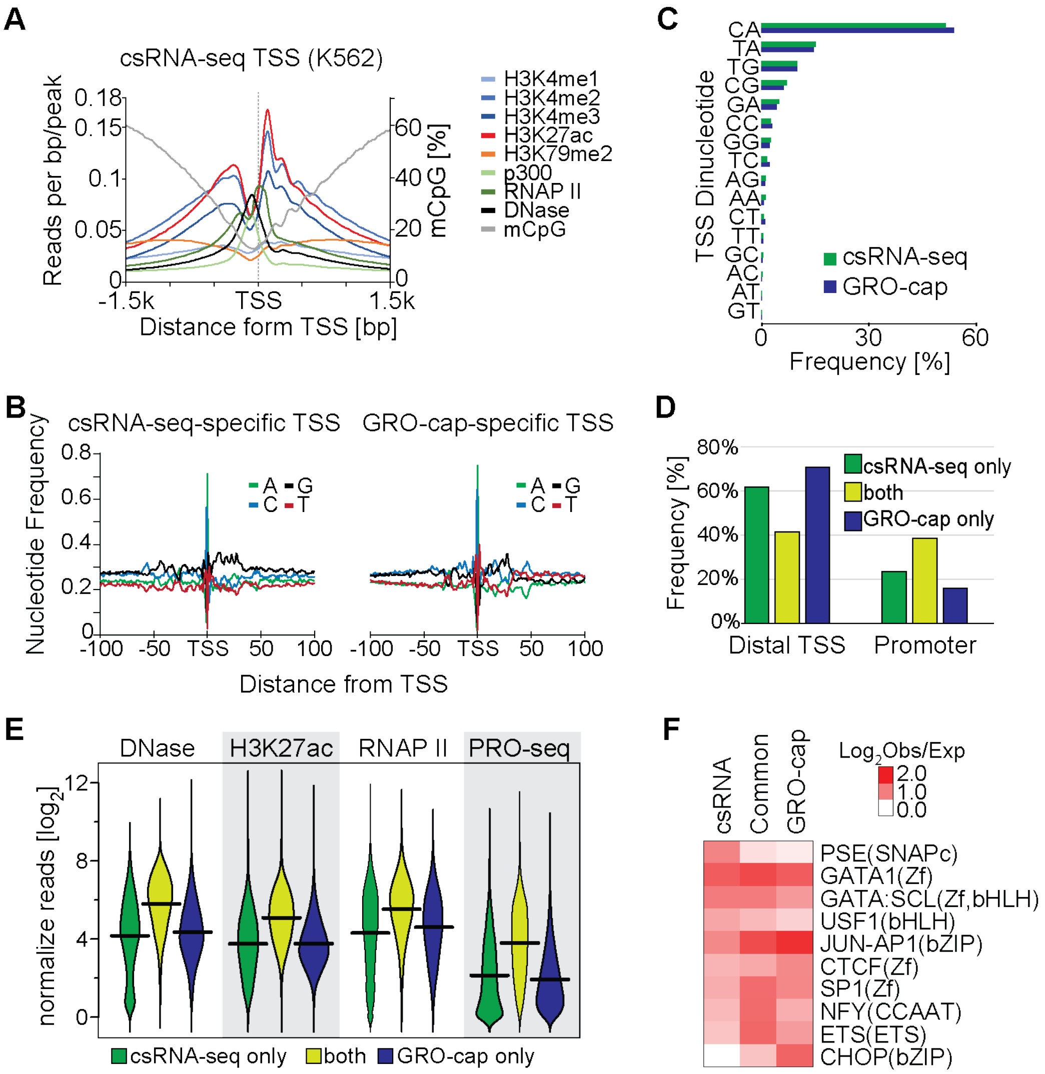

Supplemental Fig 3. csRNA-seq accurately captures initiated stable and unstable RNAs from total RNA. *(A)* Histogram of open chromatin, epigenetic marks and selected factors for all csRNA-seq TSS in K562 cells. *(B)* TSS nucleotide frequencies of GRO-cap or csRNA-seq -specific TSS. *(C)* Dinucleotide frequency of csRNA-seq and GRO-cap TSS (-1,+1) at the primary site of initiation in each TSS cluster. *(D)* Frequency of TSS that map to RefSeq annotated promoters or distal regions of TSS captured by only csRNA-seq, GRO-cap and both methods. *(E)* Violin plot showing the levels of open chromatin (DNaseI), H3K27 acetylation, and RNA polymerase II [-300,+300] and strand specific PRO-seq read density [-100,+300]. *(F)* DNA motif analysis of unique and shared TSS defined by GRO-cap and csRNA-seq.

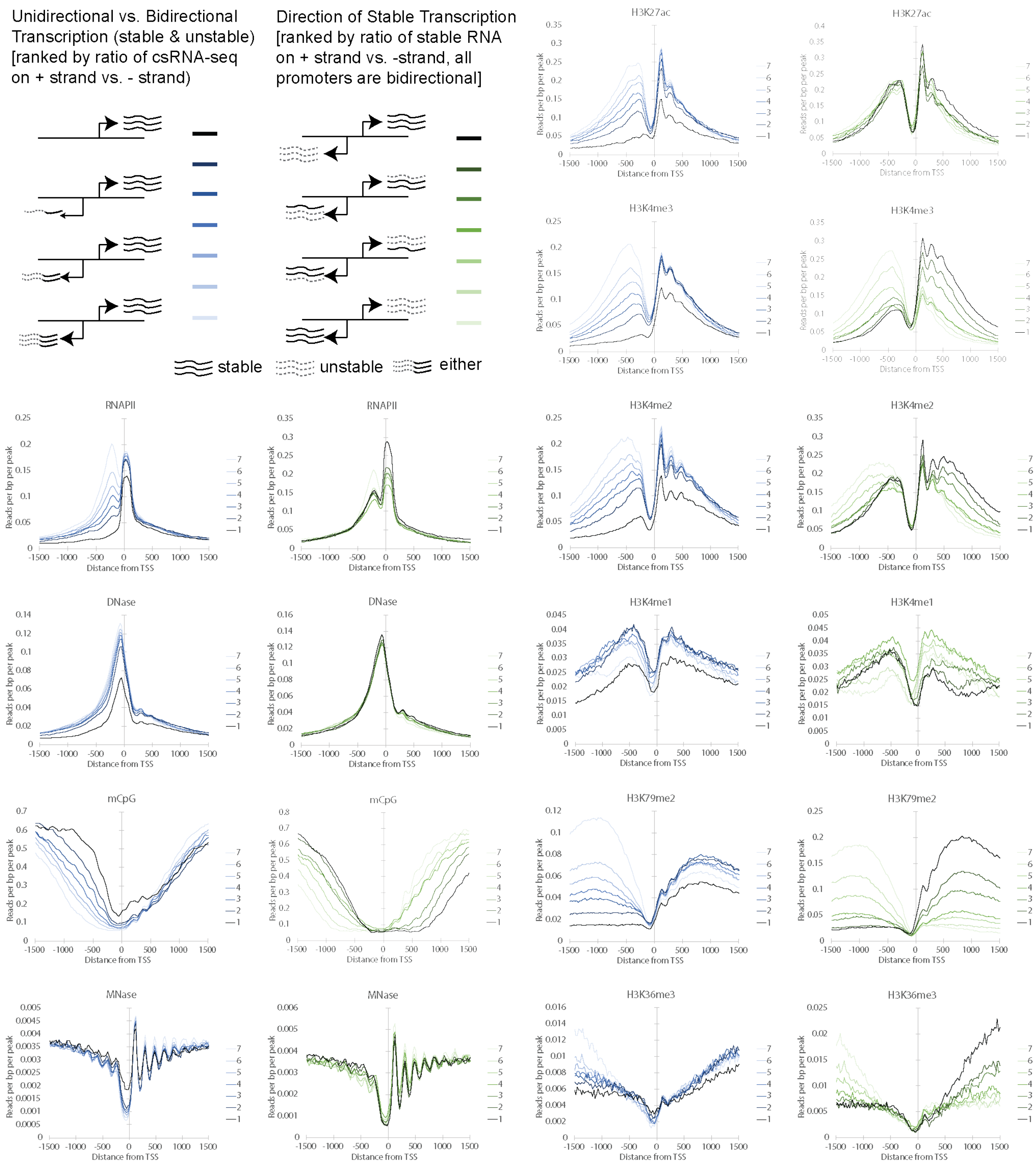

Supplemental Fig 4. Comparison of chromatin features relative to the direction of transcription (blue) or the direction of stable transcription (green). To determine directional TSS profiles, TSS with high levels of expression (>50 normalized csRNA-seq reads) were ranked by the ratio of antisense (- strand, -500,+100) to sense (+ strand, -75 to +75) csRNA-seq reads and separated into seven groups. For all groups the forward TSS is strongly expressed, while the relative strength of the antisense TSS increases as the blue color becomes lighter. For stable TSS profiles, TSS with high levels of expression and divergent expression (>50 normalized csRNA-

Supplemental Fig 4 (continued). seq in both directions) were sorted by the difference in $\log_2$ ratio of RNA-seq to csRNA-seq reads between forward and antisense TSS. All TSS in this group are strongly initiated in both sense and antisense directions, but the relative stability of initiated transcripts shifts from sense to antisense as the green color becomes lighter.

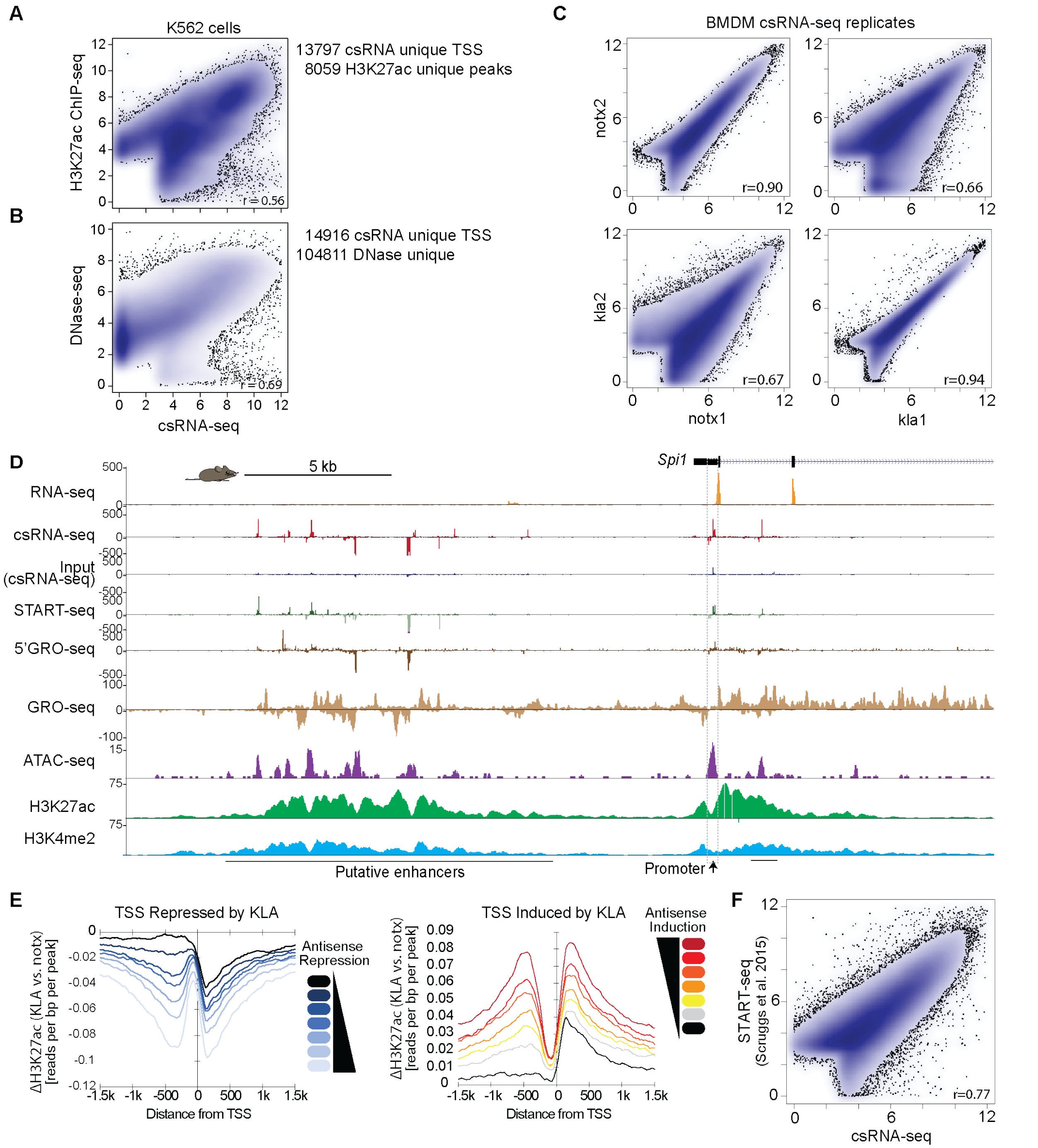

Supplement Fig 5. csRNA-seq captured changes in the transcriptome with high fidelity. Scatterplots comparing read densities of H3K27ac ChIP-seq *(A)* and DNaseI-seq *(B)* peaks with csRNA-seq TSS clusters in K562 cells. *(C)* Cross comparisons of the csRNA-seq replicates from mouse (C57) bone marrow derived macrophages (BMDMs) in resting and activated state after stimulation for 1h with KLA depicting the relative variance between replicates and experimental conditions. *(D)* UCSC genome browser shot of the *Spi1* gene in murine BMDMs 1h post KLA treatment (Chr2:91,076,768- 91,106,259). *(E)* Relative changes in the levels of

H3K27ac at antisense transcripts upon repression and induction of the sense transcript. *(F)* Comparison of Start-seq (nuclear RNA) and csRNA-seq (total RNA) in resting BMDMs.

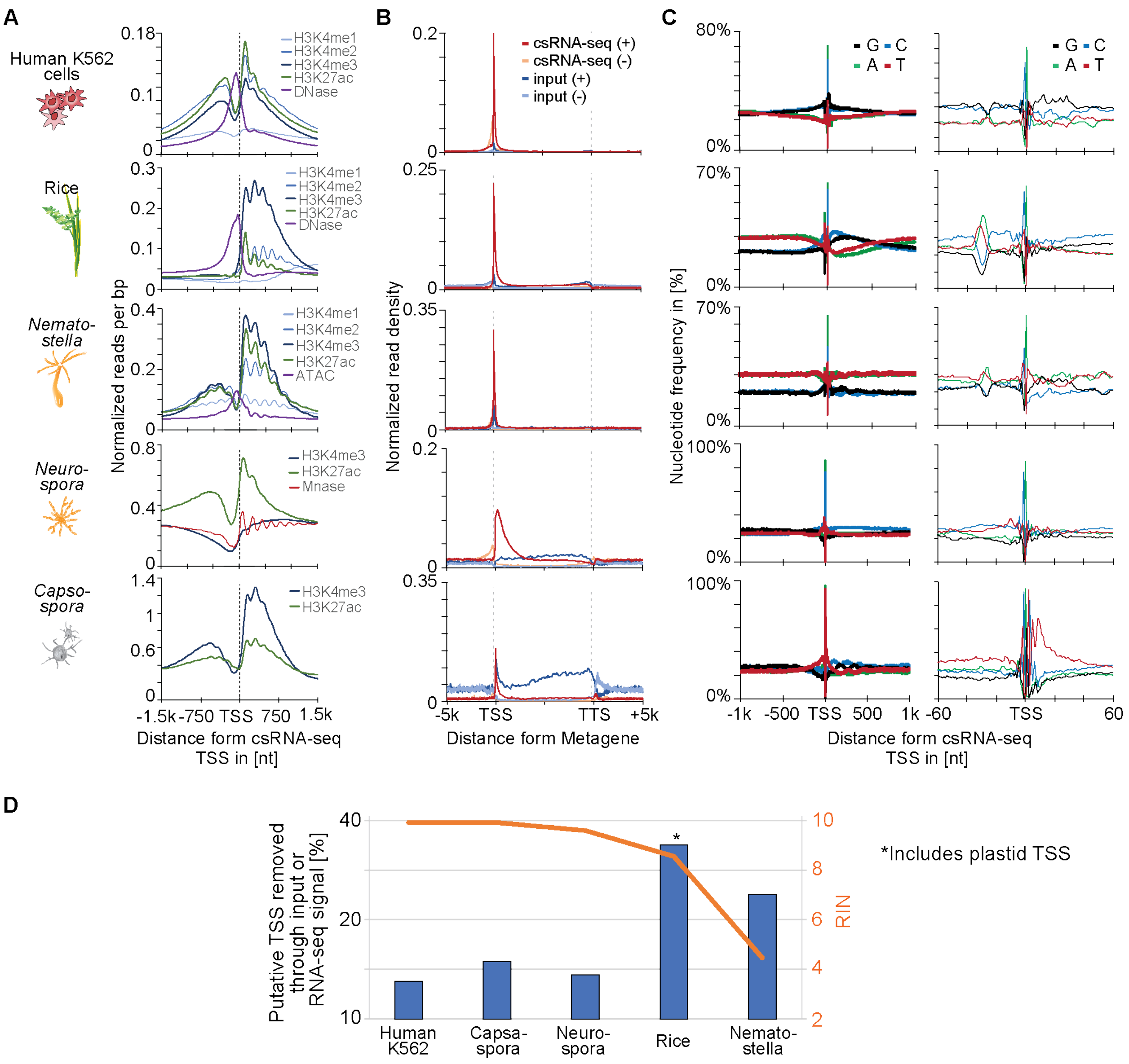

Supplemental Fig 6. csRNA-seq captures stable and transient initiating transcription across eukaryotic specimen and tissues. *(A)* Histogram of nucleosome modifications and open chromatin aligned by the csRNA-seq TSS (dotted line) determined for the five eukaryotes. *(B)* Strand-specific metagene plot summarizing where the distribution of csRNA-seq and small RNA input reads relative to existing gene annotations. *(C)* Nucleotide frequencies ± 1kb and -60 to +60 bp relative to the TSS. *(D)* Comparison of RNA quality (RIN#) and the percentage of putative csRNA-seq TSS clusters removed due to poor enrichment relative to input or RNA-seq libraries. Note: plants contain additional plastid genomes (chloroplasts, granula) that are

transcribed by prokaryotic and viral polymerases whose transcripts are uncapped and often short.

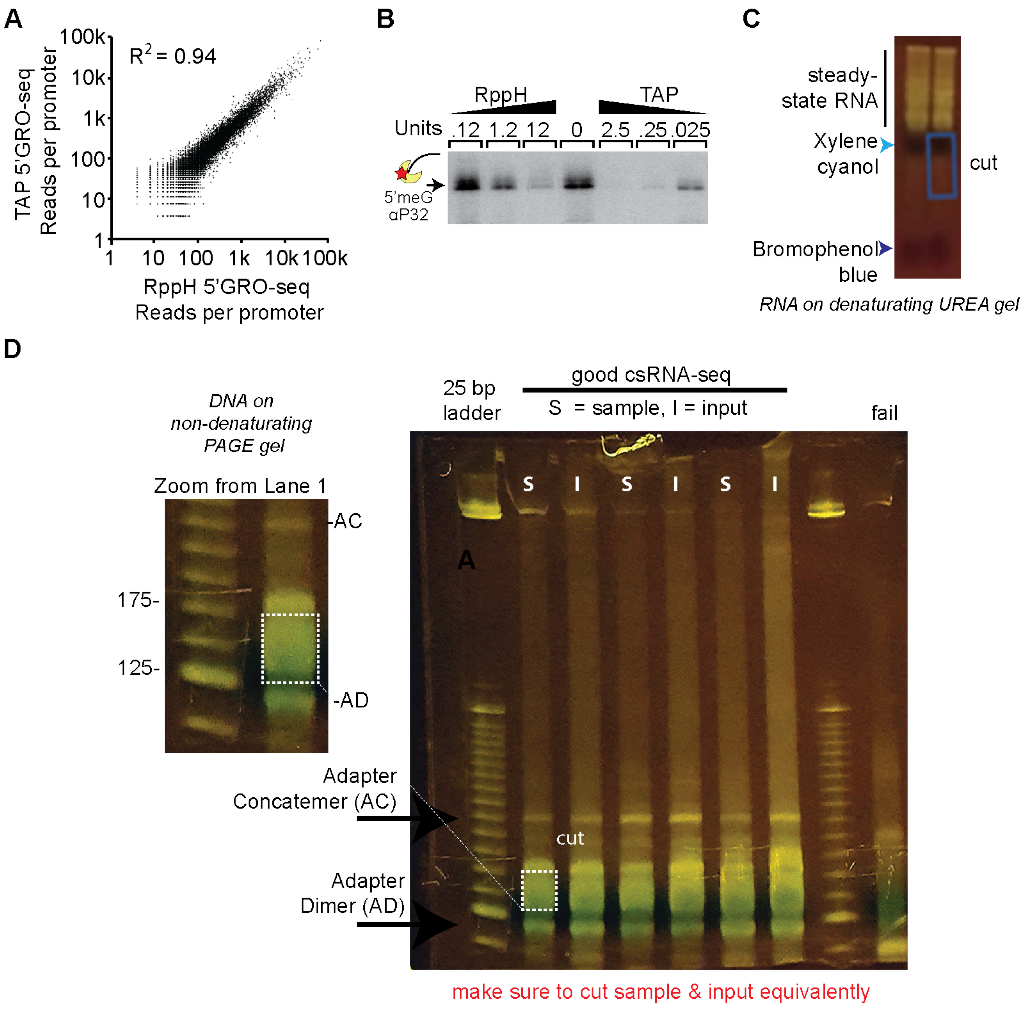

Supplement Fig 7. Additional csRNA-seq protocol details. *(A)* Correlation of TSS called from 5'GRO-seq libraries where RNAs were decapped using TAP or RppH. *(B)* Urea gel image of 150 pmol p32 capped RNA (264 nt) incubated with various amounts of TAP or RppH for 30 minutes. 1U RppH removed ~10 pmol 5'meG caps (equating 900 ng RNA). *(C)* Example gel of RNA size selection - 10 µg RNA were loaded on a 15% acrylamide 1xTBE 7M Urea gel and stained with SYBR Green for 5 minutes. *(D)* Example gel of DNA size selection on 10% acrylamide 1xTBE with a 25bp ladder (prominent band = 125 bp).

Supplementary Methods

Capped small RNA-seq

Rationale: sequencing short and capped small RNAs enriches for initiated RNA polymerase II transcripts and provides acute transcription start sites (TSS) at single nucleotide resolution.

Background: capped small RNA-seq (csRNA-seq) is an adaptation of Start-seq (Scruggs et al. 2015; Nechaev et al. 2010) and small RNA sequencing methods (Lister et al. 2009; Gu et al. 2012; Seila et al. 2008; Project and Affymetrix/Cold Spring Harbor Laboratory ENCODE Transcriptome Project 2009). Total or nuclear RNA can serve as input for csRNA-seq. Nuclear RNA is enriched for the desired transcripts but it is critical that transcription and the relative presentation of cell types is not affected during the nuclear isolation procedure. Using total RNA as input enables its application to a wide variety of samples including single celled organisms, plants, fungi as well as fresh, frozen or otherwise preserved tissue and RNA. csRNA-seq accurately captures initiated transcripts, and while they are a good proxy for gene expression, they may not reflect the final steady-state RNA or protein levels in the sample.

In this study we aim to enrich for small 5'-capped RNAs generated by RNA polymerase II. However, by altering the enzymes, csRNA-seq could also be applied to specifically enrich for RNA species with other 5' modifications (e.g. (Liu et al. 2018)). To enrich for 5'-capped RNAs, monophophorylated RNA is first degraded by 5'-monophosphate-dependent RNase (Term51020, Epicentre or XRN1, New England Biolabs [NEB]). Subsequently, RNA is globally dephosphorylated using Calf Intestinal Alkaline Phosphatase (CIP, NEB). As CIP does not remove the 5'-Cap, subsequent treatment of the sample with 5´ Pyrophosphohydrolase (RppH) removes pyrophosphates such as the 5' cap to leave a 5' monophosphate RNA which is substrate for 5'-monophosphate-dependent 5'-Adapter ligation by T4 RNA ligase 1. The results of using RppH are highly similar to using Tobacco acid phosphatase (r=0.94, Fig. S7A) although the activity per Unit differs drastically (1U RppH removed ~10 pmol 5'meG caps (equating 900 ng RNA); Fig. S7B). It is important to note that CIP does not readily remove 2',3'-cyclic phosphates that result from RNA fragmented by E1 elimination, most RNases or ribozymes (Honda et al. 2016). Good RNA quality improves the analysis due to minimal false TSS derived from fragmented steady-state RNAs (in part as cyclic phosphates can be spontaneously eliminated by protons). Nevertheless, the use of input libraries to eliminate false positives (see csRNA-seq TSS cluster identification) allowed us to successfully call TSS from csRNA-seq libraries generated from highly degraded total RNA (RIN < 2).

csRNAs-seq website

A complete protocol as well as the analysis tutorial and updates on csRNA-seq are available at http://homer.ucsd.edu/homer/ngs/csRNAseq/

csRNAs-seq Protocol

Any method that yields quality total RNA can be used for RNA isolation. Caution needs to be taken with some column-based kits to ensure all small RNAs are captured effectively. About 5-25 µg total RNA is typically used as starting material (~2-5 million cells; ~2-5 mm^3 of tissue). The upper limit is primarily determined by the gel's capacity. However, small RNAs enrichment prior to gel purification can be performed using SPRIselect beads in combination with NaCl/PEG and Isopropanol precipitation (Beckman Coulter protocol IB-18479A) thereby significantly increasing the relevant input material.

We recommend LowBinding tubes and tips for the protocol.

A) Size selection:

1. Pre-run 15% acrylamide, 8M UREA and 1x TBE gel (homebrew or Invitrogen EC6885BOX with 7M UREA) gel 20-30 minutes in 1x TBE at 200V or using heat control (55°C).
2. Mix 5-25 µg total RNA in up to 15 µl TE'T (10 mM Tris-HCl pH 7.5, 0.1 mM EDTA, 0.05% Tween 20) with an equal volume of FLB (95% Formamide, 0.005% bromophenol blue, 0.005% xylene cyanol, 1 mM EDTA) in a total volume of no more than 30 µl.
3. Denature RNA mixture on 75°C heat block for 5 minutes, then quickly place on wet ice.
4. Flush out the wells of the pre-run gel and the load the RNA with *generous* spacing. Do not load side by side. Run gel ~40 minutes at 200 V (or heat controlled) until the bromophenol blue dye is ¾ (or about 8 cm) down.
    a. Meanwhile: for each sample, puncture a 0.5 ml tube 3x with a 22 gauge needle and place it onto a 1.5 ml low binding Eppendorf tube. Label both tubes. This setup will be used to shredder the gel. Keep everything sterile.
5. Stain the gel in 1x TBE + ethidium bromide (0.5 µg/ml final) and excise RNA. Cut bottom: 20 nt (or halfway between bromophenol blue and xylene cyanol) and the just under the first visible sharp band (varies between species and tissues: 55-75 nt, Fig. S7C). Note: you may not see any stained RNA in the area at all. That's ok! Place the gel slice into the punctured 0.5 ml tube. RNA ladders can be used to exactly cut 20 nt - top but it's not necessary.
6. Shredder the gel slice using the setup from 4a and centrifugation at ~20k g for 2 minutes. Depending on the rotor, you may want to label the side of the tube and incrementally increase the speed to not lose the tube's lid.
7. To the gel slurry, add 300 µl GEB (0.4 M NaOAc pH 5.5, 10 mM Tris pH 7.5, 1 mM EDTA, 0.05% Tween 20) and elute RNA for about 2 hours under gentle agitation at RT. An RNase inhibitor may be added if not well trained in RNA work.
8. Transfer the mixture into a spin filter column (Millipore UFC30HVNB or smaller). Cut the connection to the collection tube and place the column onto a new clean low-binding 1.5 µl Eppendorf tube. Spin for 2 minutes at 1000g. Add 1.5 µl GlycoBlue (AM9516, Thermo Fisher Scientific), mix and then 3 volumes of 99-100% ethanol. Mix well and precipitate at -80°C overnight or in liquid N_2 for 30 minutes.

9. Pellet RNA by centrifugation for 30 minutes at 4°C in a microcentrifuge at >21k g. Remove the supernatant, quickspin to remove the remainder and wash the RNA pellet once with 75% ethanol. Completely remove the ethanol, quick spin once more and remove all remaining liquid using a gel loading tip. Allow the pellet to air-dry for 2-3 minutes. *-- optional stop point --*

B) 5' cap selection (1st):

1. Resuspend the RNA pellet in 6 µl TE'T. Heat the 1.5 ml tube to 75°C for 2 minutes, then quickly place on ice for 2 minutes. This step is critical to linearize miRNAs and other sRNAs with secondary structures.
2. **Important: take a 10 % input control at this point**. Add 0.6 µl 'unselected input small RNA' to a PCR strip that contains 1 µl TE'T to ease accurate transfer.
3. To B1, add 14 µl Terminator MM (1x: 10.75 µl dH_2O+0.05%Tween 20 [dH_2O+0.05%T], 2 µl Terminator Buffer A, 0.25 µl RNase Inhibitor, 1 µl Ter51020 (Epicentre)) and incubate at 30°C for one hour.
   ** it is recommended to test CIP/rSAP for RNase (see the troubleshooting section) prior to use **
4. To each reaction, add 30 µl CIP1 MM (1x: 24 µl dH_2O+0.05%T, 5 µl CutSmartBuffer, 1 µl CIP (or equivalent units of rSAP/QuickCIP), mix and incubate at 37°C for an additional 45 minutes.
5. To clean up the sample add 1 vol of beads (Agencourt A63987, or 2 µL SpeedBeads in 20% PEG8000, 2.5M NaCl, test for RNase if homebrew). Mix well.
6. Then add 1 total volume of 100% isopropanol and incubate on ice for 10 minutes.
7. Collect beads on a magnet, remove supernatant. Wash beads 2x with 200 µl 80% EtOH +0.05%Tween.
8. Spin to collect all remaining EtOH, remove and air dry beads.

C) 2nd 5' cap selection: (This step can be omitted but provides cleaner results)

1. Resuspend RNA in 25 µl TET. Heat the 1.5 ml tube to 75°C for 3 minutes, then place on ice.
2. To each reaction, add 25 µl CIP2 MM (1x: 18.5 µl dH_2O+0.05%T, 5 µl CutSmartBuffer, 0.5 µl RNase Inhibitor, 1 µl CIP), mix and incubate at 37°C for 30-45 minutes.
3. Add 100 µl TE'T and collect beads on magnet.
4. Mover supernatant to a new tube 1.5 ml tube and TRIzol purify RNA (add 500 µL TRIzol, 5' vortex, then 140 µl $CHCl_3$, mix, spin 12,000g for 10 minutes at RT! to obtain cleaner interphase).
5. Transfer SN, add 1/10th volume NaOAc and 0.5 µL GlycoBlue, precipitate in 1 volume 100% isopropanol (Never place in -80°C°C!). Spin down 30' ~20k g at 4°C.
6. Wash pellet in 75% EtOH and transfer the pellet in 75% EtOH to PCR tube with a fresh p1000 µl pipette tip.
7. Quickspin and remove all EtOH and dry pellet in the PCR tube, strip or 96 well plate.

-- optional stop point --

D) Library preparation

Note: Library preparation can also be done as described in Hetzel *et al.* 2016. After the RppH step, one can also follow any small RNA library prep kit i.e. NEB e7330 or other commercial kits to ease the procedure. To expedite the process, we pipette the master mix into new PCR strip lids. After incubation, exchange the lids to add the new master mix to the samples by thorough inversion.

1. Dissolve from C.7 (5' cap-enriched) RNA pellets in 3 µl dH_2O+0.05%T. Heat to 75°C for 90 seconds in a thermocycler, then place on ice.
2. In parallel, heat input samples from B.2 to 75°C for 90 seconds in a thermocycler, then place on ice. You can generate your small RNA input libraries exactly the same as your csRNA-seq libraries from here on. However, to save reagents, we generate libraries with 50% vol of each mastermix as the csRNA-seq libraries. If your feel uncomfortable with pipetting small volumes, input libraries can be prepared exactly the same as the csRNA-seq ones.
3. To decap RNA, add 5 µl RppH MM (0.8 µl T4RNA Ligase buffer [NEB], 3 µl PEG8000 [NEB], 0.3 µl RNase Inhibitor, 1 µl RppH [NEB]), *mix well* by thorough inversion flics and incubate at 37°C for 1 hour. (Use 2.5 µl [50%] for input if you feel comfortable with the small volumes).
4. Place on ice for 2 minutes, then quickspin.
5. For 3' Adapter ligation add 4 µl 3'-MM (0.4 µl T4RNA Ligase buffer, 0.4 µl dH_2O+0.1%T, 1 µl PEG8000, 0.2 µl 10 µM denaturated 3'Adapter, 1 µl T4RNA ligase 2 KQ [NEB]). Mix well by manual shaking. Quickspin. Incubate at 22°C for 1-2h. We recommend adding the 3'-MM to a fresh PCR strip lids and then just exchange the lids for faster progress.
6. RT-primer hybridization. Add 1 µl 5 µM RT primer, incubate at 75°C for 3 minutes, then 37°C for 20 minutes and 25°C for 5 minutes.
7. For 5' Adapter ligation, add 4 µl 5'-MM (0.5 µl T4RNA Ligase buffer, 1.9 µl dH_2O+0.1%T, 0.5 µl PEG8000, 0.2 µl RNase Inhibitor, 0.2 µl 100 mM ATP, 0.2 µl 10 µM denaturated 5'Adapter, 0.5 µl T4RNA ligase1 [NEB]).
8. For reverse transcription, add 5.25 µl RT MM (4.5 µl 5x First Strand Buffer [NEB 7421Z] which contains dNTPs and DTT, 0.75 µl reverse transcriptase) and incubate at 50°C for 1 hour.
   -- Optional stop point --
9. To each sample, add 30.5 µl PCR MM (27.5 µl 2x PCR MM (i.e. M0287L), 0.2 µl 100 mM forward barcode primer, 2.8 µl 5M Betaine) and 2 µl 10 µM reverse barcode primer. Denaturate at 94˚C for 3 minutes then cycles at 94˚C 45 seconds, 63˚C for 30 seconds and 70˚C 15 seconds. To finish, hold at 70˚C for 5 minutes to ensure completion. We usually need between 11-14 cycles. You may use qPCR to estimate the range of linear amplification.

   -- Optional stop point --

E) DNA selection and sequencing

1. PCR purification: to D9, add 100 µl Beads MM (2 µl EDTA-washed Sera-Mag Magnetic beads, 48 µl 5M NaCl, 50 µl 40% PEG). Vortex well and precipitate for 10 minutes.
2. Collect beads on a magnet and remove supernatant.
3. Wash beads twice with 80% ethanol + 0.5% Tween
4. Remove all residual ethanol and let it dry well.
5. Elute in 15 µl 1x DNA gel loading buffer.
   For gel purification, it is recommended to run the "input" adjacent to the csRNA-seq sample to ensure both libraries are size selected for the same lengths.

6. Size select libraries on a 12-well 10% acrylamide 1x TBE gel with 0.5 µl 25 bp ladder (Invitrogen or alternatives with 125 and 175bp bands) and run gel till xylene cyanol is about 1 cm from running off (Fig.S7D).
7. Meanwhile:
   a. For each sample, puncture a 0.5 ml tube 3x with a 22 gauge needle and place it onto a 1.5 ml low binding Eppendorf tube.
   b. Prepare DNA cleanup columns for each sample using Zymogen ChIP DNA Clean & Concentrator (D5205, with caps).

   Shredder the gel slice by centrifugation at max speed, then add 150 µl DNA-GEB (0.5M LiCl, 0.1% LDS, 5 mM EDTA, 10 mM Tris pH 7.5) and elute under gentle agitation for a few hours or O/N.

8. Add 750 µl DNA binding buffer directly to the gel/DNA-GEB slurry, mix, then transfer the whole slurry onto the concentrator column. Close caps.
9. Spin columns for 3 minutes at 2000g, then 1 minute at 8000g till all liquid is in the flow thru.
10. Wash column once with 200 µl Zymo Wash Buffer (containing EtOH).
11. Invert column and flick out the gel slury.
12. Repeat wash with 200 µl Zymo Wash Buffer (containing EtOH) and continue according to the manufacturer's recommendation.
13. Elute DNA using 10-20 µl Sequencing-TE'T (10 mM Tris *pH 8.0*, 0.1 mM EDTA, 0.05% Tween 20). (Repeated elution increases the yield approximately 15% but reduces the final concentration.)
14. Samples were quantified by Qbit (Invitrogen) using the hsDNA mode and sequenced using the Illumina NextSeq 2500 platform for 75 cycles single end.

csRNA-seq troubleshooting:

- **I do not see any small RNA on my UREA gel -** It's not a bad sign if you don't see a small RNA smear but strong RNA bands at higher molecular weight. If you can readily see a smear, it is usually due to lower RNA quality. You may wait for a slight smear to show up after 5-10 minutes or use a stronger stain such as SYBR Gold, but take caution(!) as more sensitive stains make cutting the higher limit more difficult. See Fig.S7.

- **Do I have RNase contaminations? -** You can check your starting reagents as well as each step in the protocol by adding a small proportion (1-2 µl or 10%) to a PCR strip with sterile TE'T. Add IDT's RNase Alert substrate (#11-04-03-03) and incubate at 37˚C for 15 minutes to hours. Using a negative control (TE'T) and a highly diluted concentration(s) of RNase will enable you to assess all your reagents or steps during the protocol.

- **I have too many adapter dimers or none!** Adapter dimers can have two main sources: too little material (RNA) or insufficient annealing of the RT primer to the 3'Adapter prior to 5'Adapter ligation by T4 RNA ligase 1. When working with small input quantities (i.e. <1 µg) consider reducing the amount of adapters to 1 pg or less. Less adapters will give you more final product. If you have sufficient input material, check the concentration of your RT primer and the annealing step.

- **miRNAs, too many miRNAs!** Yes, these little fellows are the reason why we CIP twice. miRNAs love to form secondary structures and are poorly depleted by Terminator or XRN1. For their depletion, it is critical to heat the sample to 75˚C and quickly cool down to omit secondary structures.

- **0,05% Tween!** Although we use low binding tubes, avoid that sRNAs stick to tubes by making sure to always maintain 0.05% tween in your reactions.

csRNA-seq TSS cluster identification

TSS clusters, defined as clusters of transcription initiation sites spaced close to one another, were found using HOMER's *findcsRNATSS.pl* tool that automates the following analysis steps to produce an annotated list of likely TSS from the experiment. First, to find an initial set of putative TSS regions, 150 bp regions composed of clusters of strand-specific csRNA-seq reads were identified across the genome the same way that ChIP-seq peaks are identified (Heinz et al. 2010). Only peaks with a minimum read-depth of 7 reads per 10^7 aligned reads and had 2-fold more reads per bp than the surrounding 10 kb were considered for further analysis, eliminating loci with minimal numbers of supporting reads or regions with high levels of diffuse signal.

In order to eliminate regions that are likely to come from contaminates or other regions not representative of transcription initiation, short RNA input libraries (and/or total RNA-seq) can

be used to further filter putative TSS regions to remove false positives. We have identified three general types of contaminants in csRNA-seq data (Fig. 1C). The first is high-abundance short RNAs (such as miRNAs or rRNA fragments) that, despite our 5' cap enrichment strategies, may still be detectable in the final csRNA-seq library at reduced levels (e.g. Supplementary Fig. S1D). The second source comes from highly expressed/abundant RNA transcripts that can yield an increased density of small RNAs along their mature transcript lengths (i.e. usually on exons, Supplementary Fig. S1C,E). These transcript-associated short RNAs are likely technical degradation/fractionation products that arise during RNA extraction or during sample handling since their relative levels are usually higher in samples with degraded RNA (i.e. lower RIN values). The third source of contamination comes from short capped RNAs that are stable within the cell, such as snRNAs and snoRNAs that contain a 5' cap structure and are short enough to be included in the RNA size selection step. One important confounding factor that complicates the analysis is that the relative contribution of each of these contaminants in the csRNA-seq and small RNA input libraries can vary depending on the organism or specific cell type analyzed, RNA quality, or the specific size range of RNA extracted at the beginning of the csRNA-seq protocol. For example, if a small RNA input sample has several very highly expressed miRNAs, the relative number of reads from small RNAs representing the degradation products along highly expressed genes may be reduced relative to a sample with relatively few highly expressed miRNAs.

To determine the appropriate enrichment thresholds to compare the ratios of csRNA-seq to control libraries (small RNA input or RNA-seq data), we adopted a semi-supervised approach based on expectations from existing genome annotations. First, putative TSS regions are annotated based on their sense-strand overlap with annotated TSS and exons from known transcript descriptions. For this study, we used the official ENSEMBL annotated gene annotations associated with each species genome. For all annotated TSS- and exon-associated putative TSS clusters, we calculate the ratio of csRNA-seq to control libraries and identify the optimal ratio that discriminates the positive detection of TSS regions (likely true positives) while limiting the identification of downstream exon associated putative TSS (likely false positives). By default, only multi-exon/spliced genes are used in this analysis. The optimal threshold is defined as the ratio that generates the largest difference in cumulative distributions of putative TSS regions in annotated TSS regions relative to downstream exons. This ratio is then applied to all putative TSS clusters (regardless of annotation) to identify the final set of valid TSS clusters. This supervised threshold detection approach is most needed when RNA quality is low and increasing numbers of csRNA-seq reads map to highly-expressed exons. When filtering TSS based on RNA-seq signal, RNA-seq reads are quantified from [-150,-10] relative to the TSS to avoid removing valid TSS of highly expressed transcripts that may result in a high density of RNA-seq reads just downstream of the TSS. Putative TSS that exceed the optimized thresholds compared to small RNA input and/or RNA-seq controls are then reported as the final set of TSS clusters. Reported genomic coordinates for each TSS cluster are centered on the primary TSS (i.e. mode) in the cluster.

To estimate the likely stability of transcripts initiating from each TSS, total RNA-seq reads (sense strand) are quantified from [-100,+500] relative to the TSS. "Stable TSS" were defined as TSS clusters containing at least 2 per 10^7 RNA-seq reads within this region. Bidirectional or divergent transcription for a given TSS cluster was calculated by quantifying

csRNA-seq signal on the opposite strand [-500,+100] relative to the TSS. Regions with at least 2 csRNA-seq reads per 10^7 were called as 'bidirectional' TSS. TSS clusters were further annotated based on their overlaps with annotated gene regions (i.e. exons, introns, etc.) and the closest annotated gene promoters where also identified to assess their distal annotation (promoter-distal TSS defined as >500 bp from annotated gene TSS).

It is difficult to distinguish if csRNA-seq TSS clusters found in the vicinity of genes represent true mRNA transcript TSS or if they initiate from enhancers or other promoter-distal regulatory elements that do not directly lead to transcription of the gene. To distinguish gene TSS from other regulatory elements, we looked for evidence that stable transcripts encoding the gene emanate from a given TSS. TSS clusters located within 200 bp of an annotated transcript 5' end on the correct strand were considered as gene TSS. To allow for the identification of gene TSS that may be associated with novel transcript isoforms, we performed an unbiased transcriptome assembly of the RNA-seq data using *Stringtie* (Pertea et al. 2015) using default parameters, only considering newly discovered transcript isoforms that share exon structure with annotated genes as determined by *gffcompare* (class codes '=' and 'j'). The TSS was considered successfully recovered for a given gene if a csRNA-seq TSS was found at the 5' end for at least one of the annotated (Ensembl) or de novo (Stringtie) transcripts associated with that gene (i.e. Supplemental Fig. S2F).

Analysis of changes in enhancers vs. nearby gene expression

To compare changes in distal regulatory elements among csRNA-seq, H3K27ac ChIP-seq, and ATAC-seq, we calculated the induction of gene expression based on GRO-seq data from untreated (Ctrl.) and KLA-stimulated macrophages (Link et al. 2018) along the full gene body (>0.25 FPKM). Promoter-distal csRNA-seq TSS or H3K27ac/ATAC-seq peaks were annotated to the nearest expressed gene within 200 kb but greater than 3 kb away. Log_2 fold change ratios were calculated for enhancers and genes by adding a pseudocount of 3 reads per 10^7 to both numerator and denominator to avoid divide by zero calculations, and correlation coefficients were calculated using $\log_2$ ratios (KLA/Ctrl.).

Analysis of transcription directionality, stability, and regulation with respect to histone modifications

Gradient plots depicting the distribution of epigenetic features relative to TSS were calculated for TSS clusters with high levels of transcription (>50 csRNA-seq reads per 107) to avoid the inclusion of weaker TSS that may have reduced signal associated with epigenetic modifications. To assess bidirectional transcription, TSS were then sorted based on the ratio of csRNA-seq sense [-75,+75] to antisense reads [-500,+100]. This list was then partitioned into 7 groups and the distribution of various NGS experiments were plotted for each group (Fig. S4).

The directionality of stable transcription was calculated for TSS clusters that were strongly transcribed bidirectionally (>50 csRNA-seq reads per 10^7 in both sense and antisense directions) where at least one of the directions contained evidence for stable transcripts (>2

RNA-seq reads per 10^7). The stability ratio of both sense and antisense regions were then calculated by finding the $\log_2$ ratio of RNA-seq (stable) to csRNA-seq reads (transcription). These values were subtracted and this value was used to sort and partition the TSS into 7 groups to reveal TSS with extreme bias in stable transcription to one direction or the other.

To partition directionally regulated TSS clusters from KLA stimulated macrophages (Fig. S5E), we first limited the analysis to either significantly induced or downregulated TSS clusters. TSS were then sorted based on the difference in csRNA-seq signal (KLA vs. Ctrl) detected in the antisense direction [-500,+100] and partitioned into 7 groups. Then distribution of H3K27ac signal in Ctrl and KLA conditions were calculated and subtracted to derive the changes in H3K27ac for each group.

RppH assay

150 pmol p32 capped RNA (264 nt) incubated with TAP or RppH for 30 minutes, stopped by addition of 2 mM EDTA and 1 vol formamide on ice and subsequently separated by UREA gel electrophoresis. Gels were imaged and quantified on a phosphorimager. 1U RppH removed ~10 pmol 5'meG caps (equating 900 ng RNA). RNA was synthesized by T7 in vitro transcription and capped using the Vaccinia Capping System (NEBM2080) and αP32-GTP.

Supplementary Table 1. Comparison of the mapping stats and fraction of stable vs. unstable transcription start sites among the 5' enriched sequencing data analyzed.

	Sequencing Reads [Mio]			TSS clusters				Directionality [%]					
File	csRNA	input	RNAseq	Total	Distal	Stable	Bidirectional	SS	SU	S	US	UU	U
K562 csRNA-seq TSS	36.3	32.5	15.2	53903	46.6%	34.8%	77.9%	9.5%	21.5%	3.7%	19.9%	27.0%	18.4%
K562 GRO-cap TSS	32.1	13.0	15.2	69455	53.8%	29.1%	85.5%	7.7%	19.7%	1.7%	20.5%	37.7%	12.8%
K562 CoPRO TSS	14.4	18.2	15.2	54829	40.0%	33.4%	89.2%	9.8%	22.1%	1.5%	25.4%	31.9%	9.3%
HCT116 csRNA-seq TSS	17.8	13.1	65.6	59671	48.9%	26.4%	82.5%	6.3%	18.9%	1.2%	16.3%	41.0%	16.3%
H9 csRNA-seq TSS	13.3	13.6	42.9	63436	46.1%	29.0%	79.3%	6.1%	19.9%	3.0%	19.4%	33.9%	17.7%
BMDM notx csRNA-seq TSS	24.3	19.7	64.0	55369	53.3%	19.3%	89.0%	2.8%	15.3%	1.1%	14.1%	56.7%	10.0%
BMDM KLA csRNA-seq TSS	23.0	27.8	57.2	46407	52.0%	20.4%	86.3%	2.8%	15.7%	2.0%	13.6%	54.4%	11.7%
BMDM notx Start-seq TSS	28.7	N/A		73216	59.0%	16.4%	86.3%	2.3%	12.9%	1.2%	12.3%	58.8%	12.5%
Capsaspora csRNA-seq TSS	7.9	1.8	8.3	6533	14.5%	96.7%	47.8%	42.5%	3.8%	50.3%	1.4%	0.1%	1.9%
Neurospora csRNA-seq TSS	36.1	8.3	13.3	16032	41.4%	76.2%	78.0%	23.7%	34.8%	17.8%	11.0%	8.6%	4.3%
Nematostella csRNA-seq TSS	3.4	1.3	31.6	17670	53.7%	82.3%	34.0%	23.4%	6.7%	52.2%	2.8%	1.1%	13.9%
Rice csRNA-seq TSS	41.2	13.5	10.3	33522	35.5%	59.5%	38.4%	3.1%	15.5%	40.9%	10.1%	9.8%	20.6%

S = stable
U = unstable

Supplementary Table 2. Overview of the analyzed published data and original datasets generated in this study.

Sample Name	Assay	Organism	Cell Type	Accession	Reference	PMID
K562 csRNA-seq r1	csRNA-seq	Homo sapiens	K562	n/a	this study	this study
K562 csRNA-seq r2	csRNA-seq	Homo sapiens	K562	n/a	this study	this study
K562 csRNAinput r1	sRNA input	Homo sapiens	K562	n/a	this study	this study
K562 csRNAinput r2	sRNA input	Homo sapiens	K562	n/a	this study	this study
K562 total RNA-seq	total RNA-seq	Homo sapiens	K562	n/a	this study	this study
HCT116 csRNA-seq r1	csRNA-seq	Homo sapiens	HCT116	n/a	this study	this study
HCT116 csRNA-seq r2	csRNA-seq	Homo sapiens	HCT116	n/a	this study	this study
HCT116 csRNAinput r1	sRNA input	Homo sapiens	HCT116	n/a	this study	this study
HCT116 csRNAinput r2	sRNA input	Homo sapiens	HCT116	n/a	this study	this study
H9 csRNA-seq r1	csRNA-seq	Homo sapiens	H9	n/a	this study	this study
H9 csRNA-seq r2	csRNA-seq	Homo sapiens	H9	n/a	this study	this study
H9 csRNAinput r1	sRNA input	Homo sapiens	H9	n/a	this study	this study
H9 csRNAinput r2	sRNA input	Homo sapiens	H9	n/a	this study	this study
BMDM notx csRNA-seq r1	csRNA-seq	Mus musculus	Macrophage	n/a	this study	this study
BMDM notx csRNA-seq r2	csRNA-seq	Mus musculus	Macrophage	n/a	this study	this study
BMDM notx csRNAinput r1	sRNA input	Mus musculus	Macrophage	n/a	this study	this study
BMDM notx csRNAinput r2	sRNA input	Mus musculus	Macrophage	n/a	this study	this study
BMDM KLA1h csRNA-seq r1	csRNA-seq	Mus musculus	Macrophage	n/a	this study	this study
BMDM KLA1h csRNA-seq r2	csRNA-seq	Mus musculus	Macrophage	n/a	this study	this study
BMDM KLA1h csRNAinput r1	sRNA input	Mus musculus	Macrophage	n/a	this study	this study
BMDM KLA1h csRNAinput r2	sRNA input	Mus musculus	Macrophage	n/a	this study	this study
Nematostella csRNA-seq r1	csRNA-seq	Nematostella vectensis	Whole organism	n/a	this study	this study
Nematostella csRNA-seq r2	csRNA-seq	Nematostella vectensis	Whole organism	n/a	this study	this study
Nematostella csRNAinput r1	sRNA input	Nematostella vectensis	Whole organism	n/a	this study	this study
Nematostella csRNAinput r2	sRNA input	Nematostella vectensis	Whole organism	n/a	this study	this study
Neurospora csRNA-seq r1	csRNA-seq	Neurospora crassa	Whole organism	n/a	this study	this study
Neurospora csRNA-seq r2	csRNA-seq	Neurospora crassa	Whole organism	n/a	this study	this study
Neurospora csRNA-seq r3	csRNA-seq	Neurospora crassa	Whole organism	n/a	this study	this study
Neurospora csRNAinput r1	sRNA input	Neurospora crassa	Whole organism	n/a	this study	this study
Neurospora csRNAinput r2	sRNA input	Neurospora crassa	Whole organism	n/a	this study	this study
Neurospora total RNA-seq	total RNA-seq	Neurospora crassa	Whole organism	n/a	this study	this study
Rice csRNA-seq r1	csRNA-seq	Oryza sativa	Leaves	n/a	this study	this study
Rice csRNA-seq r2	csRNA-seq	Oryza sativa	Leaves	n/a	this study	this study
Rice csRNA-seq r3	csRNA-seq	Oryza sativa	Leaves	n/a	this study	this study
Rice csRNAinput r1	sRNA input	Oryza sativa	Leaves	n/a	this study	this study
Rice csRNAinput r2	sRNA input	Oryza sativa	Leaves	n/a	this study	this study
Rice total RNA-seq	total RNA-seq	Oryza sativa	Leaves	n/a	this study	this study
Capsaspora csRNA-seq r1	csRNA-seq	Capsaspora owczarzaki	Whole organism	n/a	this study	this study
Capsaspora csRNAinput r1	sRNA input	Capsaspora owczarzaki	Whole organism	n/a	this study	this study
Capsaspora total RNA-seq	total RNA-seq	Capsaspora owczarzaki	Whole organism	n/a	this study	this study

Supplemental Table S2 (continued)

**Public Transcriptomic Data:**	**Assay**	**Organism**	**Cell Type**	**Accession**		**PMID**
K562 GRO-cap	GRO-cap	Homo sapiens	K562	SRR1552480	Core et al. 2014	25383968
K562 GRO-cap input	GRO-cap(input)	Homo sapiens	K562	SRR1552481	Core et al. 2014	25383968
K562 CoPRO	CoPRO	Homo sapiens	K562	SRR7458419, SRR7458420, SRR7458425	Tome et al. 2018	30349116
K562 CoPRO input	CoPRO(input)	Homo sapiens	K562	SRR7458421, SRR7458422, SRR7458426	Tome et al. 2018	30349116
Hct116 total RNA-seq	total RNA-seq	Homo sapiens	HCT116	SRR2969247-SRR2969249	Lee et al. 2016	26724866
Hct116 PRO-seq	PRO-seq	Homo sapiens	HCT116	SRR6290531-SRR6290534, SRR6290539-SRR6290542, SRR6290547-SRR6290550, SRR6290555-SRR6290558	Rao et al. 2017	28985562
Hct116 nuclear RNA-seq (ctrl)	nuc RNA-seq	Homo sapiens	HCT116	SRR7909090, SRR7909092	Davidson et al. 2019	30840897
Hct116 nuclear RNA-seq (DIS3 depleted)	nuc RNA-seq	Homo sapiens	HCT116	SRR7909091, SRR7909093	Davidson et al. 2019	30840897
H9 total RNA-seq	total RNA-seq	Homo sapiens	H9	SRR6225384, SRR6225385	Fiddes et al. 2018	29856954
BMDM notx GRO-seq	GRO-seq	Mus musculus	Macrophage	SRR6660228, SRR6660229	Link et al. 2018	29779944
BMDM KLA1h GRO-seq	GRO-seq	Mus musculus	Macrophage	SRR6660226, SRR6660227	Link et al. 2018	29779944
BMDM notx RNA-seq	RNA-seq	Mus musculus	Macrophage	SRR6660267, SRR6660268	Link et al. 2018	29779944
BMDM KLA1h RNA-seq	RNA-seq	Mus musculus	Macrophage	SRR6660261, SRR6660262	Link et al. 2018	29779944
BMDM notx 5'GRO-seq	5'GRO-seq	Mus musculus	Macrophage	SRR6936828	Link et al. 2018	29779944
BMDM KLA1h 5'GRO-seq	5'GRO-seq	Mus musculus	Macrophage	SRR6936827	Link et al. 2018	29779944
BMDM notx Start-seq	Start-seq	Mus musculus	Macrophage	SRR1605862, SRR1605863	Scruggs et al. 2015	26028540
BMDM LPS30m Start-seq	Start-seq	Mus musculus	Macrophage	SRR1605864, SRR1605865	Scruggs et al. 2015	26028540
BMDM LPS2h Start-seq	Start-seq	Mus musculus	Macrophage	SRR1605866, SRR1605867	Scruggs et al. 2015	26028540
Nematostella total RNA-seq	total RNA-seq	Nematostella vectensis	Whole organism	SRR836055, SRR836050	Schwaiger et al. 2014	24642862

Supplemental Table S2 (continued)

**Public Epigenomics Data:**	**Assay**	**Organism**	**Cell Type**	**Accession**		**PMID**
K562 DNase-seq	DNase-seq	Homo sapiens	K562	ENCFF001DUI, ENCFF001DUJ, ENCFF162DLQ, ENCFF370IOC, ENCFF543XCF, ENCFF715ZAU	Thurman et al. 2012	22955617
K562 H3K27ac	ChIP-seq	Homo sapiens	K562	SRR227385, SRR227386	ENCODE Project Consortium 2012	22955616
K562 H3K4me3	ChIP-seq	Homo sapiens	K562	ENCFF010SAE, ENCFF894KBP	ENCODE Project Consortium 2012	22955616
K562 H3K4me2	ChIP-seq	Homo sapiens	K562	ENCFF000BYA, ENCFF000BYF	ENCODE Project Consortium 2012	22955616
K562 H3K4me1	ChIP-seq	Homo sapiens	K562	ENCFF000VDU, ENCFF000VDV	ENCODE Project Consortium 2012	22955616
K562 H3K79me2	ChIP-seq	Homo sapiens	K562	ENCFF000BYO, ENCFF000BYS	ENCODE Project Consortium 2012	22955616
K562 H3K36me3	ChIP-seq	Homo sapiens	K562	ENCFF000BXO, ENCFF000BXR	ENCODE Project Consortium 2012	22955616
K562 RNAPII	ChIP-seq	Homo sapiens	K562	ENCFF000RWV, ENCFF000RXB, ENCFF272EEG, ENCFF959SCN	ENCODE Project Consortium 2012	22955616
K562 MNase-seq	MNase-seq	Homo sapiens	K562	SRR3211681, SRR3211682, SRR490054-SRR490064	Heidari et al. 2014, Mieczkowski et al. 2016	25228660, 27151365
K562 BS-seq	BS-seq	Homo sapiens	K562	ENCLB542OXH, ENCLB742NWU	ENCODE Project Consortium 2012	22955616
HCT116 DNase-seq	DNase-seq	Homo sapiens	HCT116	ENCFF001DCK, ENCFF001DCL	Thurman et al. 2012	22955617
HCT116 H3K27ac	ChIP-seq	Homo sapiens	HCT116	SRR6164278	Rao et al. 2017	28985562
HCT116 H3K4me3	ChIP-seq	Homo sapiens	HCT116	SRR6164280	Rao et al. 2017	28985562
H9 DNase	DNase-seq	Homo sapiens	H9	SRR412245-SRR412251	Neph et al. 2012	22955618
H9 H3K27ac	ChIP-seq	Homo sapiens	H9	SRR067373	Rada-Iglesias et al. 2011	21160473
BMDM notx ATAC-seq	ATAC-seq	Mus musculus	Macrophage	SRR6660215, SRR6660216	Link et al. 2018	29779944
BMDM KLA1h ATAC-seq	ATAC-seq	Mus musculus	Macrophage	SRR6660213, SRR6660214	Link et al. 2018	29779944
BMDM notx H3K27ac	ChIP-seq	Mus musculus	Macrophage	SRR6660232, SRR6660233	Link et al. 2018	29779944

Supplemental Table S2 (continued)

**Public Epigenomics Data:**	**Assay**	**Organism**	**Cell Type**	**Accession**		**PMID**
BMDM KLA1h H3K27ac	ChIP-seq	Mus musculus	Macrophage	SRR6660230, SRR6660231	Link et al. 2018	29779944
BMDM KLA1h H3K4me2	ChIP-seq	Mus musculus	Macrophage	SRR6660234, SRR6660235	Link et al. 2018	29779944
Nematostella H3K27ac	ChIP-seq	Nematostella vectensis	Whole organism	SRR836003-SRR836007, SRR837781	Schwaiger et al. 2014	24642862
Nematostella H3K4me3	ChIP-seq	Nematostella vectensis	Whole organism	SRR836025-SRR836030	Schwaiger et al. 2014	24642862
Nematostella H3K4me2	ChIP-seq	Nematostella vectensis	Whole organism	SRR836019-SRR836024	Schwaiger et al. 2014	24642862
Nematostella H3K4me1	ChIP-seq	Nematostella vectensis	Whole organism	SRR836015-SRR836018	Schwaiger et al. 2014	24642862
Nematostella ATAC-seq	ATAC-seq	Nematostella vectensis	Whole organism	SRR6502904-SRR6502909	Sebé-Pedrós et al. 2018	29856957
Nematostella p300	ChIP-seq	Nematostella vectensis	Whole organism	SRR836041-SRR836044	Schwaiger et al. 2014	24642862
Neurospora H3K27ac	ChIP-seq	Neurospora crassa	Whole organism	SRR7690284	Bicocca et al. 2018	30468429
Neurospora H3K4me3	ChIP-seq	Neurospora crassa	Whole organism	SRR8064955-SRR8064958	Zhu et al. 2019	31068130
Neurospora MNase-seq	MNase-seq	Neurospora crassa	Whole organism	SRR3181964, SRR3181965, SRR3181968, SRR3181969	Seymour et al. 2016	27172195
Rice H3K27ac	ChIP-seq	Oryza sativa	Leaves	SRR3213601	Fang et al. 2016	27558448
Rice H3K4me3	ChIP-seq	Oryza sativa	Leaves	SRR6955804, SRR6955805	Qi et al. 2018	29931324
Rice H3K4me2	ChIP-seq	Oryza sativa	Leaves	SRR094790	Zhang et al. 2012	22110044
Rice H3K4me1	ChIP-seq	Oryza sativa	Leaves	SRR6955793, SRR6955794	Qi et al. 2018	29931324
Rice DNase	DNase-seq	Oryza sativa	Leaves	SRR094106-SRR094108	Zhang et al. 2012	22110044
Capsaspora H3K27ac	ChIP-seq	Capsaspora owczarzaki	Whole organism	SRR2120277, SRR2120278, SRR2120287, SRR2120286, SRR2120268, SRR2120269	Sebé-Pedrós et al. 2016	27114036
Capsaspora H3K4me3	ChIP-seq	Capsaspora owczarzaki	Whole organism	SRR2120275, SRR2120284, SRR2120266	Sebé-Pedrós et al. 2016	27114036
Capsaspora ATAC	ATAC-seq	Capsaspora owczarzaki	Whole organism	SRR2120282, SRR2120283, SRR2120291, SRR2120273, SRR2120274	Sebé-Pedrós et al. 2016	27114036

Supplemental References

Bicocca VT, Ormsby T, Adhvaryu KK, Honda S, Selker EU. 2018. ASH1-catalyzed H3K36 methylation drives gene repression and marks H3K27me2/3-competent chromatin. *Elife* **7**. http://dx.doi.org/10.7554/eLife.41497.

The ENCODE Project Consortium. 2012. An integrated encyclopedia of DNA elements in the human genome. *Nature* **489**: 57–74.

Fang Y, Wang X, Wang L, Pan X, Xiao J, Wang X-E, Wu Y, Zhang W. 2016. Functional characterization of open chromatin in bidirectional promoters of rice. *Sci Rep* **6**: 32088.

Fiddes IT, Lodewijk GA, Mooring M, Bosworth CM, Ewing AD, Mantalas GL, Novak AM, van den Bout A, Bishara A, Rosenkrantz JL, et al. 2018. Human-Specific NOTCH2NL Genes Affect Notch Signaling and Cortical Neurogenesis. *Cell* **173**: 1356–1369.e22.

Gu W, Lee H-C, Chaves D, Youngman EM, Pazour GJ, Conte D Jr, Mello CC. 2012. CapSeq and CIP-TAP identify Pol II start sites and reveal capped small RNAs as C. elegans piRNA precursors. *Cell* **151**: 1488–1500.

Heidari N, Phanstiel DH, He C, Grubert F, Jahanbani F, Kasowski M, Zhang MQ, Snyder MP. 2014. Genome-wide map of regulatory interactions in the human genome. *Genome Res* **24**: 1905–1917.

Heinz S, Benner C, Spann N, Bertolino E, Lin YC, Laslo P, Cheng JX, Murre C, Singh H, Glass CK. 2010. Simple combinations of lineage-determining transcription factors prime cis-regulatory elements required for macrophage and B cell identities. *Mol Cell* **38**: 576–589.

Honda S, Morichika K, Kirino Y. 2016. Selective amplification and sequencing of cyclic phosphate-containing RNAs by the cP-RNA-seq method. *Nat Protoc* **11**: 476–489.

Lee S, Kopp F, Chang T-C, Sataluri A, Chen B, Sivakumar S, Yu H, Xie Y, Mendell JT. 2016. Noncoding RNA NORAD Regulates Genomic Stability by Sequestering PUMILIO Proteins. *Cell* **164**: 69–80.

Lister R, Pelizzola M, Dowen RH, Hawkins RD, Hon G, Tonti-Filippini J, Nery JR, Lee L, Ye Z, Ngo Q-M, et al. 2009. Human DNA methylomes at base resolution show widespread epigenomic differences. *Nature* **462**: 315–322.

Liu W, Duttke SH, Hetzel J, Groth M, Feng S, Gallego-Bartolome J, Zhong Z, Kuo HY, Wang Z, Zhai J, et al. 2018. RNA-directed DNA methylation involves co-transcriptional small-RNA-guided slicing of polymerase V transcripts in Arabidopsis. *Nat Plants* **4**: 181–188.

Mieczkowski J, Cook A, Bowman SK, Mueller B, Alver BH, Kundu S, Deaton AM, Urban JA, Larschan E, Park PJ, et al. 2016. MNase titration reveals differences between nucleosome occupancy and chromatin accessibility. *Nat Commun* **7**: 11485.

Nechaev S, Fargo DC, dos Santos G, Liu L, Gao Y, Adelman K. 2010. Global analysis of short RNAs reveals widespread promoter-proximal stalling and arrest of Pol II in Drosophila. *Science* **327**: 335–338.

Neph S, Vierstra J, Stergachis AB, Reynolds AP, Haugen E, Vernot B, Thurman RE, John S, Sandstrom R, Johnson AK, et al. 2012. An expansive human regulatory lexicon encoded in transcription factor footprints. *Nature* **489**: 83–90.

Pertea M, Pertea GM, Antonescu CM, Chang T-C, Mendell JT, Salzberg SL. 2015. StringTie enables improved reconstruction of a transcriptome from RNA-seq reads. *Nat Biotechnol* **33**: 290–295.

Affymetrix ENCODE Transcriptome Project; Cold Spring Harbor Laboratory ENCODE Transcriptome Project.. 2009. Post-transcriptional processing generates a diversity of 5′-modified long and short RNAs. *Nature* **457**: 1028–1032.

Qi M, Li Z, Liu C, Hu W, Ye L, Xie Y, Zhuang Y, Zhao F, Teng W, Zheng Q, et al. 2018. CGT-seq: epigenome-guided de novo assembly of the core genome for divergent populations with large genome. *Nucleic Acids Res* **46**: e107.

Rada-Iglesias A, Bajpai R, Swigut T, Brugmann SA, Flynn RA, Wysocka J. 2011. A unique chromatin signature uncovers early developmental enhancers in humans. *Nature* **470**: 279–283.

Scruggs BS, Gilchrist DA, Nechaev S, Muse GW, Burkholder A, Fargo DC, Adelman K. 2015. Bidirectional Transcription Arises from Two Distinct Hubs of Transcription Factor Binding and Active Chromatin. *Mol Cell* **58**: 1101–1112.

Sebé-Pedrós A, Saudemont B, Chomsky E, Plessier F, Mailhé M-P, Renno J, Loe-Mie Y, Lifshitz A, Mukamel Z, Schmutz S, et al. 2018. Cnidarian Cell Type Diversity and Regulation Revealed by Whole-Organism Single-Cell RNA-Seq. *Cell* **173**: 1520–1534.e20.

Seila AC, Mauro Calabrese J, Levine SS, Yeo GW, Rahl PB, Flynn RA, Young RA, Sharp PA. 2008. Divergent Transcription from Active Promoters. *Science* **322**: 1849–1851.

Seymour M, Ji L, Santos AM, Kamei M, Sasaki T, Basenko EY, Schmitz RJ, Zhang X, Lewis ZA. 2016. Histone H1 Limits DNA Methylation in Neurospora crassa. *G3* **6**: 1879–1889.

Thurman RE, Rynes E, Humbert R, Vierstra J, Maurano MT, Haugen E, Sheffield NC, Stergachis AB, Wang H, Vernot B, et al. 2012. The accessible chromatin landscape of the human genome. *Nature* **489**: 75–82.

Tome JM, Tippens ND, Lis JT. 2018. Single-molecule nascent RNA sequencing identifies regulatory domain architecture at promoters and enhancers. *Nat Genet* **50**: 1533–1541.

Zhang W, Wu Y, Schnable JC, Zeng Z, Freeling M, Crawford GE, Jiang J. 2012. High-resolution mapping of open chromatin in the rice genome. *Genome Res* **22**: 151–162.

Zhu Q, Ramakrishnan M, Park J, Belden WJ. 2019. Histone H3 lysine 4 methyltransferase is required for facultative heterochromatin at specific loci. *BMC Genomics* **20**: 350.
